# A systematic review of the research progress of traditional Chinese medicine against pulmonary fibrosis: from a pharmacological perspective

**DOI:** 10.1186/s13020-023-00797-7

**Published:** 2023-08-03

**Authors:** Shanbo Qin, Peng Tan, Junjie Xie, Yongfeng Zhou, Junning Zhao

**Affiliations:** 1 Key Laboratory of Biological Evaluation of TCM Quality of State Administration of Traditional Chinese Medicine, Sichuan Academy of Chinese Medicine Sciences, Chengdu, 610041 China; 2 State Key Laboratory of Southwestern Chinese Medicine Resources, Chengdu University of Traditional Chinese Medicine, Chengdu, 611137 China

**Keywords:** Pulmonary fibrosis, Traditional Chinese medicine, Anti-inflammation, Anti-oxidation, Endoplasmic reticulum stress

## Abstract

Pulmonary fibrosis is a chronic progressive interstitial lung disease caused by a variety of etiologies. The disease can eventually lead to irreversible damage to the lung tissue structure, severely affecting respiratory function and posing a serious threat to human health. Currently, glucocorticoids and immunosuppressants are the main drugs used in the clinical treatment of pulmonary fibrosis, but their efficacy is limited and they can cause serious adverse effects. Traditional Chinese medicines have important research value and potential for clinical application in anti-pulmonary fibrosis. In recent years, more and more scientific researches have been conducted on the use of traditional Chinese medicine to improve or reduce pulmonary fibrosis, and some important breakthroughs have been made. This review paper systematically summarized the research progress of pharmacological mechanism of traditional Chinese medicines and their active compounds in improving or reducing pulmonary fibrosis. We conducted a systematic search in several main scientific databases, including PubMed, Web of Science, and Google Scholar, using keywords such as idiopathic pulmonary fibrosis, pulmonary fibrosis, interstitial pneumonia, natural products, herbal medicine, and therapeutic methods. Ultimately, 252 articles were included and systematically evaluated in this analysis. The anti-fibrotic mechanisms of these traditional Chinese medicine studies can be roughly categorized into 5 main aspects, including inhibition of epithelial-mesenchymal transition, anti-inflammatory and antioxidant effects, improvement of extracellular matrix deposition, mediation of apoptosis and autophagy, and inhibition of endoplasmic reticulum stress. The purpose of this article is to provide pharmaceutical researchers with information on the progress of scientific research on improving or reducing Pulmonary fibrosis with traditional Chinese medicine, and to provide reference for further pharmacological research.

## Introduction

Pulmonary fibrosis (abbreviated as PF) is a chronic, progressive, irreversible interstitial lung disease commonly caused by multiple etiologies and characterized by the accumulation of inflammatory cells such as macrophages, neutrophils and lymphocytes in the alveoli, the proliferation and differentiation of fibroblasts, and the development of fibrous connective tissue. Ultimately, it leads to structural changes in the patient's normal lung tissue [1, 2]. In the late stages of many pulmonary diseases, PF is a common pathological manifestation. In modern medicine, interstitial lung disease is divided into two types: secondary interstitial lung disease and idiopathic interstitial lung disease. The former has a relatively clear etiology, including silicosis, pneumoconiosis, asbestosis, radiation-induced PF, and drug-induced interstitial lung disease. In contrast, the etiology of the latter is unknown, including idiopathic pulmonary fibrosis (abbreviated as IPF), cystic fibrosis, and interstitial pneumonia with autoimmune features, among which IPF is the most important [1, 3]. Due to IPF's wide involvement, long course, high mortality rate, and other characteristics, it is also known as a tumor-like disease [2]. Unfortunately, the prognosis for patients with IPF is often unfavorable, as they typically suffer from significant lung function impairment and a reduced quality of life during recovery [4, 5]. Epidemiological surveys have shown that the prevalence and incidence of IPF is increasing year by year and is more prevalent in the elderly. The mortality rate of IPF is high, and more than half of patients with IPF who were hospitalized for acute exacerbations will die during their hospitalization, and their average survival period after diagnosis is 2 to 4 years [6–8]. Notably, following the outbreak of COVID-19 in 2019, clinical observations have identified fibrosis in the lungs of patients with COVID-19 [9–11]. If PF is not controlled promptly and effectively, it will lead to a decline in lung function and seriously affect the quality of life and life expectancy of patients [12]. At present, glucocorticoids and Immunosuppressive drug, such as pirfenidone and nifanyl, are the main clinical treatments for PF, but their clinical efficacy is not satisfactory, and these drugs are also expensive and have many side effects [13–15]. In recent years, traditional Chinese medicines (abbreviated as TCM) has made great progress in improving or reducing of PF, and has become one of the alternative therapies for clinical treatment of IPF due to its significant efficacy and few side effects [16–18]. Of particular interest is that in the battle against COVID-19, TCM has shown the advantages of high efficiency and low toxicity in lung injury caused by the novel coronavirus and in the prognosis of rehabilitation, playing an important role in blocking the progression of PF and promoting the recovery of patients [19–21]. In this review article, we focus on the research progress of TCM in improving PF from a pharmacological perspective. Firstly, we summarized the research progress on the pharmacological mechanisms related to PF. Secondly, we systematically summarized the potential active compounds in TCM that can be used to improve PF, and classified the targets of these compounds. Finally, future research directions were envisioned. The following search terms were used: IPF, PF, interstitial pneumonia, natural product, herbs. The time limit is from June 2017 to June 2023. We did a systematic search of several major scientific databases, including PubMed, Web of Science, and Google Scholar. A total of 252 articles were retrieved. At that time, the focus was on screening original experimental articles that matched the theme, totaling 184 articles. We evaluated these literature and systematically reviewed the research progress of TCM in improving PF in the past five years.

## Pathogenesis of pulmonary fibrosis

The pathogenesis of PF is not yet fully elucidated, but it is known to be caused by a variety of factors: for example, PF due to silica inhalation [22], PF induced by chemicals, such as bleomycin, paraquat [23, 24], and induced by different primary diseases [25].

Modern medicine generally agrees that fibrosis can be described as an irrational form of injury repair [26, 27]. Repeated microdamage to the alveolar epithelium is the first driving factor that induces an abnormal repair process in which persistent alveolar epithelial cell damage and repair abnormalities, proliferation of fibroblasts, and accumulation of extracellular matrix (abbreviated as ECM) lead to structural disorders in the lung and the formation of fibrosis [28–30]. In the early stages of PF, there are different influencing factors, but in the later stages of fibrosis, there are the same mechanisms of action [31, 32]. The pathogenesis of PF can be roughly divided into three stages: injury, inflammation, repair. The first stage: the lung is damaged or otherwise noxiously stimulated and fibroblasts are activated and begin to secrete ECM. This phase is disease-specific and it consists mainly of lymphocyte activation and differentiation, autoimmune and immune-mediated conditions of excessive immune response, and chronic granulomatous inflammation. This is due to the persistence of identified or unidentified antigens, or other exposures. These multiple environmental risk factors, such as smoking, occupational exposure, air pollution, toxic compounds, viral infections, can repeatedly damage alveolar cells [2, 33, 34]. The second stage: mitogen-activated protein kinase (abbreviated as MAPK) and nuclear factor kappa B (abbreviated as NF-κB) pathways are activated to promote the production of a large number of cytokines [35]. Activated fibroblasts undergo structural and phenotypic changes and produce a large amount of ECM [36]. Through paracrine, inflammatory cells, including macrophages, move to the stimulated site. T cells are activated to secrete fibrogenic growth factors, such as interleukin and tumor necrosis factor alpha (abbreviated as TNF-α) [37]. Macrophages promote the proliferation and differentiation of fibroblasts and secrete a variety of cytokines, including transforming growth factor beta (abbreviated as TGF-β) and Interleukins-1 (abbreviated as IL-1) [38]. The third phase: the injury factors persisted, resulting in repeating damage of alveolar epithelial cells. Fibroblasts continue to be activated to produce more ECM [39–41]. Cytokines continue to cause tissue inflammation and collagen overexpression, ECM deposition, the beginning of a vicious circle, and finally lead to the gradual formation of PF, the loss of lung function at the idiopathic site [42–44].

## Pulmonary fibrosis-related signaling pathways

### TGF-β/Smad

Transforming growth factor-β/Smad (TGF-β/Smad) is a pleiotropic signaling pathway that plays a key role in inflammation, wound healing, fibrosis processes such as epithelial injury, myofibroblast fibroblast proliferation and differentiation and ECM production [45, 46]. TGF-β exerts its biological activity through activation of Smad-dependent and non-dependent pathways. The Smad protein family can be divided into three categories: receptor-activated (R-Smads, including Smads 1, 2, 3, 5, and 8), general-purpose (Co-Smad, including Smad 4), and inhibitory (I-Smads, including Smad 6 and 7) [47]. The TGF-β receptor is a receptor that belongs to the group with endogenous Ser/Thr kinase activity and binds to its type I and type II receptors to form a complex that leads to phosphorylation of Smad2 and Smad3 [48]. The phosphorylated Smad2 and Smad3 then further form a complex with Smad4, which undergoes nuclear translocation, activating the expression of transcription factors downstream of Epithelial-mesenchymal transition (EMT) and promoting EMT [49]. TGF-β1 also activates MAPK, phosphoinositide 3-kinase/protein kinase B pathway, and Rho pathways, induces EMT, increases the expression of collagen, fibronectin, and tissue inhibitor of matrix metalloproteinases (TIMPs), and promotes PF. Recent studies have shown that numerous active substances of natural products can improve PF through the TGF-β/Smad signaling pathway [50].

### Nrf2/ARE

When the organism is damaged by external oxidative and chemical stimuli and other stresses, it generates corresponding self-defense responses and induces a series of protective proteins. The Nuclear Factor erythroid 2-Related Factor 2/antioxidant response element (Nrf2/ARE) pathway is a classical defensive transduction pathway that can reduce the oxidative stress damage occurring in cells [51]. Nrf2 is a key factor in the cellular oxidative stress response, with antioxidant, anti-inflammatory response and cytoprotective effects [52]. Nrf2 is a key transcription factor that is essentially expressed in oxidative stress and is present in multiple organs throughout the body, and its deletion or impaired activation directly causes changes in cellular sensitivity to stressors changes in the sensitivity of cells to stressors [53, 54]. The Nrf2/ARE pathway in the lung mainly regulates the expression of antioxidant genes, thus providing protection to lung tissue [55]. When cells are attacked by reactive oxygen species or other electrophile reagents in a state of oxidative stress, Nrf2 is uncoupled from keap1 and translocated across the membrane into the nucleus. By regulating ARE activity, it further initiates the transcription of downstream regulatory antioxidant proteins and phase II detoxification enzymes, and regulates the expression of various antioxidant genes, thereby increasing the production of antioxidant substances, reducing cellular oxidative damage and maintaining intracellular redox homeostasis, thus playing an antioxidant and anti-fibrotic role [55]. By activating the Nrf2/ARE signaling pathway, the synthesis of antioxidant proteins can be increased, and thus the body’s enhanced antioxidant capacity can be achieved to delay the progression of PF [56]. The Nrf2/ARE pathway can also mediate a variety of antioxidant enzymes and phase II detoxification enzymes to protect tissues.

### PI3K/AKT

The phosphoinositide 3-kinase/protein kinase B pathway (PI3K/AKT) is one of the central intracellular signaling pathways regulating cell growth, proliferation, motility, metabolism and survival [57, 58]. PI3K is a signal transduction enzyme that phosphorylates PI (4,5) P2 to form PI (3,4,5) P3, which is activated by tyrosine kinase receptors, G protein-coupled receptors/cytokine receptors and Ras protein-associated GD Pase receptors to promote cell proliferation, survival, adhesion, differentiation, cytoskeleton organization, etc. [59, 60]. Protein kinase B (AKT) is a serine/threonine kinase downstream of PI3k, and AKT binds to PI(3,4,5) P3 near the cell membrane to form a complex. The binding of the complex to 3-phosphoinositide-dependent protein kinase 1 promotes the phosphorylation of the PH domain at the amino acid terminus of AKT, which activates downstream factors such as hypoxia-inducible factor-1 and mammalian target of rapamycin (mTOR) to participate in cell proliferation and differentiation [59, 60]. AKT is a direct target protein downstream of PI3K, which can participate in regulating cell proliferation and metabolism, promoting fibrosis-related gene transcription and protein synthesis, and activated AKT can participate in PF by activating mTOR [61]. AKT2-deficient mice can counteract bleomycin (BLM)-induced PF and inflammation, suggesting that PI3K/AKT signaling plays an important role in IPF development [62]. In addition, activation of PI3K/AKT can be involved in the development of PF by regulating its downstream genes such as mTOR, hypoxia-inducible factor-1 and Fox family [63]. It is due to the important role of PI3K/AKT in regulating receptor-mediated signaling that targeting PI3K/AKT has become a promising strategy for the treatment of IPF.

### Wnt/β-catenin

Wnt signaling pathway can be divided into classical Wnt signaling pathway (i.e. Wnt/β-catenin signaling pathway) and non-classical Wnt signaling pathway. Among them, β-catenin is the key molecule that mediates classical Wnt signaling. Wnt proteins are a group of secreted glycoproteins expressed in a variety of tissue cells, involved in a variety of signaling pathways, and play a key role in cell differentiation, cell migration, organogenesis, stem cell self-renewal and maintenance of tissue homeostasis. β-catenin is a cytoskeletal protein that, together with E-cadherin and α-catenin, is involved in the construction of cell junctions and intercellular adhesion mechanisms, and plays an important role in maintaining the stability of the intracellular environment and signaling into the nucleus [64, 65].

The Wnt/β-catenin signaling pathway plays an important role in many pathological processes in the lung and is one of the major regulatory pathways in PF [66]. In pulmonary endothelial cells Wnt/β-catenin signaling causes a shift from perivascular fibroblasts to myofibroblast-like cells, leading to ECM accumulation and increased tissue stiffness, further promoting PF [67]. Downregulation of the Wnt signaling pathway also inhibited myofibroblast differentiation, thereby ameliorating PF lesions [68]. The Wnt/β-catenin signaling pathway was significantly activated in the IPF animal model [69], and blockade of the Wnt/β-catenin pathway was also effective in attenuating lung fibrosis in mice [70].

It has been shown that Wnt/β-catenin signaling is involved in the induction of EMT during the development of fibrosis [71]. Wnt1, Wnt3A, Wnt7B, Wnt10B, Fzd2, Fzd3 and β-catenin expression were significantly increased in lung tissues of IPF patients [72]. Wnt5A and Wnt5B ligands have been reported to exert effects on pulmonary fibroblast differentiation via TGF-β [73]. The Wnt/β-catenin pathway also interacts with the TGF-β/Smad, PI3K/AKT signaling pathway and plays an important role in the pathogenesis of IPF, and inhibition of this pathway can reduce or reverse PF [74].

### NF-κB

NF-κB is one of the major nuclear transcription factors that regulate inflammatory and immune responses, as well as a signaling pathway that is present in many cell types and closely associated with intracellular biological functions and inflammatory responses [75, 76]. In addition, NF-κB binds to fixed nucleotide sequences in the promoter regions of many genes to initiate gene transcription, which plays a crucial role in the inflammatory response, the regulation of the immune system, and cell growth [77, 78]. Five transcription factors comprise the NF-κB family: NF-κB1 (p50), NF-κB2 (p52), Rel A (p65), Rel B, and c-Rel [79]. NF-κB proteins function as dimers that bind to the κB site and affect the transcription of target genes [80]. The phosphorylation of NF-κB is responsible for activating the NF-κB signaling pathway [81]. Inflammation is now believed to be one of the factors contributing to fibrosis [82–84], and during PF, NF-κB is activated, which promotes the release of large amounts of inflammatory factors such as TNF-α, IL-1β, IL-8 and TGF-β1 [85], stimulating the proliferation of fibroblasts and the deposition of collagen fibers, thereby promoting the development of organ fibrosis [86]. Several studies [87, 88] have elucidated the crucial role of the NF-κB signaling pathway in regulating acute lung injury-induced PF. NF-κB also plays a key role in the secretion of pro-fibrotic cytokines during the progression of PF [89]. In addition to inflammation, cellular senescence is an important factor leading to fibrosis that can promote the development of IPF through a variety of mechanisms, such as senescence associated secretory phenotype (SASP), telomere dysfunction, etc. [90, 91]. The NF-κB signaling pathway is a key regulator of SASP, according to [92]. SASP has been shown to be inhibited by the inhibition or knockdown of multiple components that regulate NF-κB signaling [93].

## Cytokines related to pulmonary fibrosis

### Transforming growth factor β

Transforming Growth Factor β (TGF-β) has been implicated as a central factor in the development of PF [94–96]. TGF-β has several biological roles, such as promoting wound repair by increasing ECM deposition, inflammatory cell recruitment and fibroblast differentiation [97, 98]. TGF-β1 is currently recognized as the most critical fibrogenic factor and the most potent promoter of ECM deposition ever identified. It has been shown in the literatures that the TGF-β1/Smad signaling pathway is activated during PF [99, 100], prompting the conversion of fibroblasts to myofibroblasts and alveolar epithelial and endothelial cells to mesenchymal cells. In addition, activation of the TGF-β1/Smad signaling pathway reduces the secretion and inhibits the activity of matrix protein metallases, while increasing the synthesis and secretion of matrix metalloproteinase tissue inhibitory factor, which inhibits myofibroblast apoptosis and leads to the production of large amounts of ECM and its failure to degrade properly, allowing it to accumulate in the lung and cause PF [36, 101].

### Platelet-derived growth factor

Platelet-derived growth factor (PDGF) is a peptide-like regulatory factor stored mainly in platelets, and when PF occurs, epithelial cells, alveolar macrophages and activated platelets will secrete large amounts of PDGF [102]. PDGF is closely related to the proliferation and differentiation of lung fibroblasts [103]. PDGF promotes the formation and development of PF by promoting the proliferation, migration and aggregation of lung fibroblasts, as well as regulating the synthesis and deposition of ECM. Therefore, PDGF is known as an energizing factor for fibroblast proliferation [104, 105]. In the process of PF, PDGF is mainly produced at the site of lung tissue injury, and TGF-β1 and TNF can regulate the expression of PDGF. On the one hand, PDGF can cross the cell membrane barrier through the damaged lung epithelial cells into the alveolar mesenchyme and chemotactic mesenchymal cells; On the other hand, similar to the function of TGF-β1, it can also induce fibroblast proliferation and differentiation and stimulate fibroblasts to secrete collagen, but the mechanism of action may be different from that of TGF-β1 [106]. It has been found that increased release of PDGF is observed in the lungs of IPF patients and that blocking PDGF receptor signaling in animal models of IPF attenuates the extent of PF [107].

### Interleukins

Interleukins (IL) are a class of cytokines produced mainly by lymphocytes, monocytes or macrophages and act on a variety of cells. They are complex in structure and function and play an important role in a range of processes including immune regulation and inflammation in lung tissue [108, 109]. Thirty-eight species have been named, of which approximately one third are involved in the development of PF. During PF, IL-1, IL-4, IL-6, IL-11, and IL-13 play important roles in promoting proliferation and aggregation of pulmonary fibroblasts, ECM deposition, collagen synthesis, and lung tissue remodeling [110, 111]. Among them, IL-13 has a significant effect on tissue fibrosis. It has been shown that IL-4 and IL-13 can synergistically exert activation effects on M2 type macrophages, and the activated M2 type macrophages secrete pro-fibroblastic cytokines thereby promoting the development of fibrosis [112]. IL-7, IL-10, IL-12, and IL-18 reduce PF by inhibiting inflammatory factors and modulating immunity [113–117]. Among them, IL-10 may activate macrophages through the CCL2/CCR2 axis, causing fibroblast accumulation and eventually causing fibrous degeneration [118].

### Tumor necrosis factor-α

Tumor necrosis factor-α (TNF-α) is a multi-temporal, cellular immune defense factor produced by mononuclear macrophages. TNF-α is highly expressed in the pathological process of lung injury and can participate in the local injury and inflammatory response, leading to the aggregation of inflammatory cells, which in turn stimulates massive proliferation of lung fibroblasts and secretion of collagen. Therefore, TNF-α is one of the important indicators for clinical testing of PF. On the one hand, TNF-α is involved in the process of acute inflammatory response and inhibits the repair of lung injury by promoting apoptosis of type II alveolar cells [119]. On the other hand, TNF-α promotes the differentiation of lung resident mesenchymal stem cells into myofibroblasts [120]. In the early stages of PF, macrophages aggregate, synthesize and release large amounts of TNF-α. TNF-α stimulates a massive increase in chemotactic and adhesion molecules, creating an inflammatory cell infiltrate. Therefore, TNF-α is also known as an early response factor [121]. In addition, TNF-α can act synergistically with IL-1 to promote neutrophil activation and aggregation and regulate the inflammatory response in the early stages of PF [122]. TNF-α acts mainly through the NF-κB pathway, and its mechanism may be related to the Wnt/β-catenin signaling pathway. There are NF-κB binding sites on the transcriptional promoter region or enhancer of the TNF-α gene, and the two promote each other and jointly regulate the development of PF [123].

## Pharmacological mechanism of TCM in improving pulmonary fibrosis

**Fig. 1:**
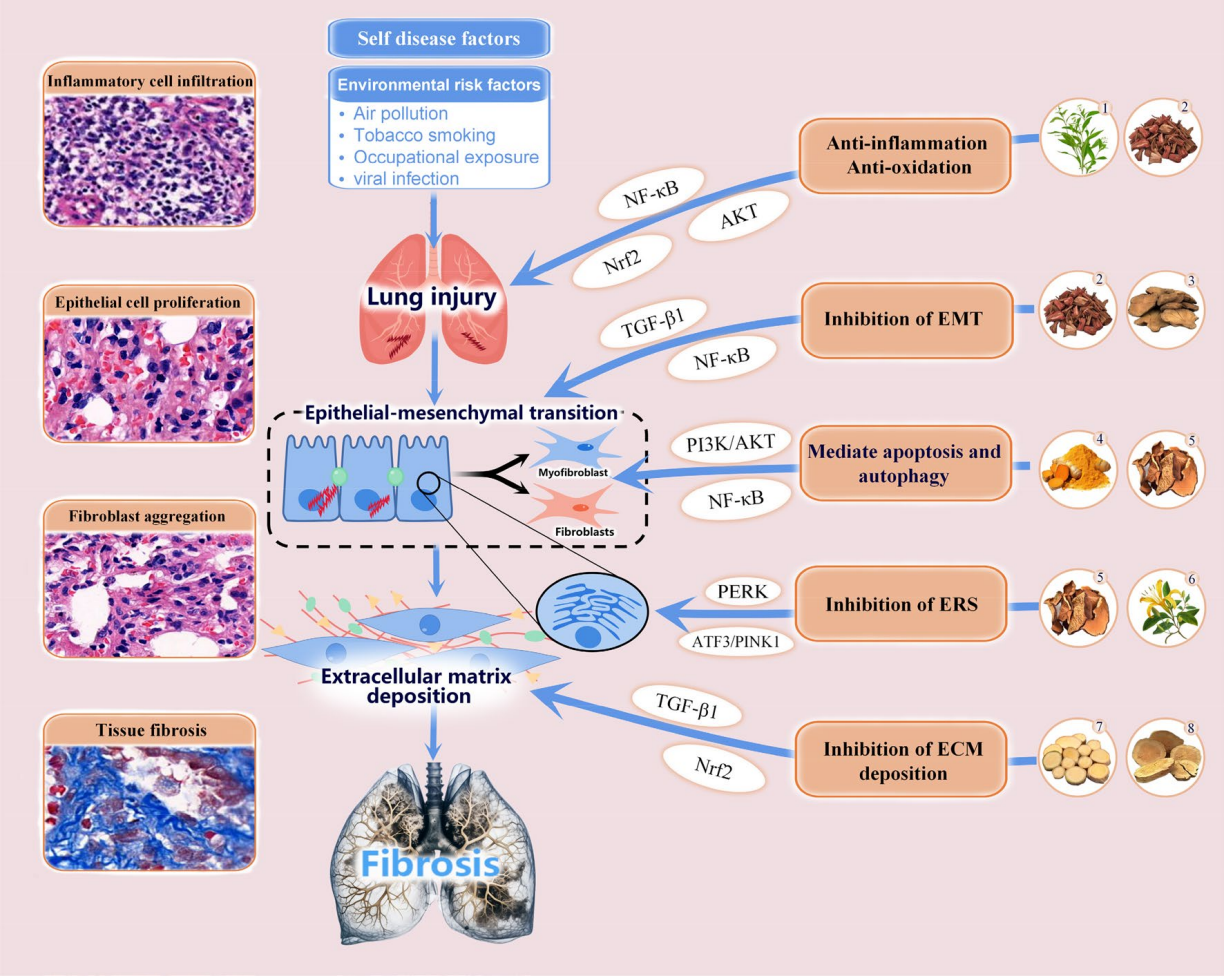
Schematic diagram of the main intervention targets of traditional Chinese medicine against pulmonary fibrosis

TCM has demonstrated significant clinical efficacy and unique advantages in improving IPF. PF is closely related with TCM terminologies such as ‘lung obstruction’, ‘lung atrophy’, and ‘lung abscess'. TCM mainly employs a dialectical treatment approach from the perspectives of tonifying the lung and kidney, invigorating the spleen and lung, and promoting blood circulation and removing blood stasis. In recent years, research on TCM therapy for PF has been increasing, and significant progress has been made in some aspects. In this section, we reviewed recent literatures and summarized that the mechanisms by which TCM improving PF can be roughly divided into five categories: inhibiting EMT, anti-inflammatory and anti-oxidative stress, improving ECM deposition, mediating cell apoptosis and autophagy, and inhibiting endoplasmic reticulum stress (ERS), a simple classification information was shown in Fig. 1.

### Inhibition of epithelial cell-mesenchymal transformation

EMT is the process by which epithelial cells lose cellular activity through a specific procedure and are then transformed into mesenchymal cells. EMT is the main source of fibroblasts and myofibroblasts in IPF [124, 125]. It has been shown that approximately 33% of myofibroblasts in the lung can be traced to cells undergoing EMT in a bleomycin-induced lung fibrosis model [126]. EMT is mainly divided into 3 types: type I EMT is mainly related to normal physiological activities of cells; type II EMT is mainly related to injury repair, tissue regeneration and organ fibrosis; type III EMT is related to tumor metastasis [127, 128]. Among them, type II EMT is mainly caused by persistent inflammation and injury, regulated by a variety of signaling factors and signaling pathways, and is an important mechanism for the occurrence and development of PF, which can be treated by inhibiting EMT progression. There is growing evidence that EMT is closely associated with fibroblast activation and PF, characterized by increased expression of α-SMA and vimentin and decreased expression of the intercellular adhesion molecule E-cadherin. Therefore, inhibition of EMT is an important way to treat PF. At present, the signaling pathways involved in EMT inhibition by TCM mainly include TGF-β/Smad, NF-κB, PI3K-Akt, and so on.

Andrographolide, a diterpenoid compound isolated from andrographis paniculata, it has pharmacological effects such as anti-inflammatory, antioxidant, and participation in regulating EMT [129, 130]. Sachin Karkale's research team found that Andrographolide can effectively reduce the expression of mesenchymal markers in PF mice and increase the expression of epithelial markers [131]. This study revealed for the first time that andrographolide could shown good anti-fibrosis activity by inhibiting inflammation and targeting EMT, which provided a new idea for the study of TCM to improve silicon induced PF. In addition to silicon induced occupational PF, andrographolide also has a certain inhibitory effect on bleomycin induced IPF. The research results of Li Jingpei's team shown that andrographolide could improve BLM induced PF in rats, and explore its mechanism from different perspectives. First, andrographolide could improve PF by inhibiting the proliferation of fibroblasts and the differentiation of myofibroblasts. This process is affected by TGF-β1 mediated regulation of Smad dependent and non dependent pathways [132]. Secondly, andrographide could inhibit TGF β1 in alveolar epithelial cells (AEC) by regulating the Smad2/3 and Erk1/2 signaling pathways [132]. Then, andrographolide could inhibit EMT in lung epithelial cells through AKT/mTOR signaling pathway, thereby reducing BLM induced PF [134]. These results shown that andrographis paniculata could improve PF through multiple ways and targets, which has great development potential and is worth our in-depth study.

Emodin is an anthraquinone compound with multiple biological activities isolated and purified from rhubarb. Zhou's team research shown that emodin could inhibit NE induced EMT in RLE-6TN and A549 cells, and its mechanism of action was related to NE induced Notch1 lysis [135]. This study preliminarily revealed the mechanism of emodin inhibiting EMT at the cellular level, which has certain reference value for further understanding the pharmacological effect of emodin in improving PF. Emodin can also regulated NF-κB and TGF- β1/Smad3 signaling pathway inhibits EMT and improves silica induced PF [136]. Rhapontin is another important compound in rhubarb, and Tao's team found in vitro experiments that rhapontin could activate AMPK and inhibit TGF- β/Smad pathway reversal of ECM [137]. This experiment confirmed the anti-PF activity of rhapontin for the first time. On the whole, rhubarb has important medicinal value in improving PF, but its pharmacology and toxicology still need further experimental and clinical research.

Salvia miltiorrhiza is a commonly used herbal medicine to treat cardiovascular and pulmonary diseases. Salvianolic acid B is a bioactive component extracted from Salvia miltiorrhiza, which has strong anti-inflammatory and antioxidant effects. Liu's team first confirmed the anti fibrotic activity of salvianolic acid B through a cell model, and further found in animal models that salvianolic acid B inhibits the transdifferentiation of lung fibroblasts by activating Nrf2 signaling [138]. Cryptotanshinone is a diterpenoid compound with antioxidant, anti-inflammatory, and antibacterial activities. Research has shown that CPT exhibits good anti-fibrotic effects in both in vitro and in vivo, inhibiting various cell proliferation and TGF-β induced EMT [139]. Although these research results suggest that Salvia miltiorrhiza may be a potential drug for improving IPF, these studies have only been conducted on animal or cell models and further clinical research is still needed.

In addition to the above-mentioned TCM that can improve PF, the detailed information of other TCM studies in the past five years that can improve PF by inhibiting EMT were summarized and presented in Table 1. It should be noted that the traditional efficacy of most Chinese medicines in the table is related to clearing away heat and detoxification, promoting blood circulation to remove blood stasis, relieving cough and resolving phlegm, which may suggest that the traditional efficacy is a reference factor that cannot be ignored when we are looking for natural drugs with anti PF activity.

**Table 1:** Details about some traditional Chinese medicines improving pulmonary fibrosis by inhibiting epithelial cell-mesenchymal transformation

No	Source	Genus information	Traditional efficacy	Active compound	Experiment model	Administration dose	Therapeutic target	Cytokine	Refs.
1	*Scutellariae radix*	Scutellaria	Clearing away heat and dampness, purging fire and detoxifying	Astragalus polysaccharides	BLM-induced PF mice and A549 cells	Mice (200 mg/kg)	Inhibit NF-κB signaling	TGF-β1, NF-κB, α-SMA, Collagen I, CHOP	[140]
2				Baicalin	BLM-induced PF rats and RPF	Rats (50 mg/kg)	Regulation of CaMKII and PI3K/AKT signaling	PI3K, AKT, Bax, Bcl-2	[141]
						Cell (20, 40, 60, 80 μg/mL)			
3					Radiation-induced EMT model	Cell (2, 10, 50 μmol/L)	Suppress EMT	Smad2/3, ERK, GSK3β	[142]
4				Baicalein	MRC-5 cells	Cell (1–80 μmol/L)	Inhibit miR-21	STAT3, TGF-β1, COL1 A1, α-SMA,	[143]
5				Calycosin, CA	BLM-induced PF mice and MLE-12 cells	Mice (7, 14 mg/kg)	Inhibit AKT/GSK3β/β-catenin signaling	AKT, GSK3β, β-catenin, E-cadherin	[144]
						Cell (0–80 μmol/L)			
6	Curcumae longae rhizoma	Zingiberaceae	Promoting blood circulation and removing blood stasis, Regulating menstruation and relieving pain	Curcumin	HUVEC cells	Cell (5, 10 μmol/L)	Regulation of NRF2-DDAH-ADMA-NO signaling	TGF-β1, Nrf2, DDAH, PRMT, ERK1/2	[145]
7					BLM-induced PF mice and A549 cells	Mice (75 mg/kg)	Suppress EMT	EGFR, Ki67	[146]
						Cell (20 μmol/L)			
8					A549 cells	Cell (20 μmol/L)	Inhibit TGF-β1/Smad/non Smad signaling	TGF-β1, Smad	[147]
9					A549 cells	Cell (0–1000 μmol/L)	Suppress EMT	ROS, α-SMA, TGF-β1	[148]
10					NHLF cells	Cell (10 μmol/L)	Down-regulation of hsa-miR-6724-5p expression	KLF10	[149]
11					CCD-19Lu cells	Cell (0–50 μmol/L)	activate PPARγ	TGF-β1, α-SMA, PPARγ	[150]
12					BLM-induced PF mice	Mice (75, 150 mg/kg)	Activates PPARγ and CREB signaling	PPARγ, CREB	[151]
13	*Andrographis herba*	Acanthaceae	Clearing heat and detoxification, cooling blood	Andrographolide	NIH 3T3, PLF cells	Rats (10 mg/kg)	Inhibits TGF-β1/Smad2/3 and Erk1/2 signaling	TGF-β1, Smad2/3	[132]
						Cell (2, 5, 10 μmol/L)			
14					BLM-induced PF rats and A549 cells	Rats (10 mg/kg)	Regulation of AKT/mTOR signaling	AKT, mTOR	[134]
15					Silica-induced PF mice	Mice (3, 10 mg/kg)	Inhibition of ECM precipitate formation	GSH, NF-κB, CTGF	[131]
16					A549 cells	Cell (0–20 μmol/L)	Inhibits A and B signaling	TGF-β1, Smad2/3, MMP-9	[133]
17	*Salviae miltiorrhizae*	Lamiaceae	Promoting blood circulation and removing blood stasis, reducing swelling and relieving pain	Tanshinone IIA	BLM-induced PF mice and PLFs, NIH-3T3 cells	Mice (5, 10, 20 mg/kg)	Inhibition of TGF-β1-Smad3 signaling	Nrf2, TGF-β1	[152]
						Cell (0.01–500 μmol/L)			
18				Salvianolic acid B	BLM-induced PF rats and MRC-5 cells	Rats (20 mg/kg)	Regulates Nrf2 signaling	Nrf2, α-SMA, TGF-β1	[138]
						Cell (40 μmol/L)			
19				Salvianolic acid B/sodium tanshinone IIA sulfonate	THP-1 cells	Cell (0–600 μg/mL)	Inhibit TGF-β1 signaling	TGF-β1, α-SMA	[153]
20				Cryptotanshinone	BLM-induced PF mice and A549, NIH/3T3, HPF cells	Mice (30, 60 mg/kg)	Suppress EMT		[139]
						Cell (0–20 μmol/L)			
21	Rhei radix et rhizoma	Polygonaceae	Purging and defecating, clearing heat and detoxification	Emodin	Silica-induced PF mice and A549 cells	Mice (25, 50, 100 mg/kg)	Regulation of NF-κB and TGF-β1/Smad3 signaling	NF-κB, TGF-β1	[136]
22					RLE-6TN, A549 cells	Cell (50, 100, 200 nmol/L)	Suppress EMT	Notch1, C-MYC	[135]
23				rhapontin	BLM-induced PF rats and THP-1 cells	Rats (25, 50, 100 mg/kg)	Regulation of TGF-β/Smad signaling	TGF-β, Smad	[137]
						Cell (50 μmol/L)			
24	Various plant sources			quercetin	RLE/Abca3 cells	Cell (20 μmol/L)	Regulation of Smad and b-catenin signaling	Smad, b-catenin	[154]
25					BLM-induced PF mice and HELF cells	Mice (25, 50, 100 mg/kg)	Inhibits SphK1/S1P signaling	TGF-β, SphK1, S1P	[155]
						Cell (10, 20, 40 μmol/L)			
26	Polygoni cuspidati rhizoma et radix	Polygonaceae	Clearing heat and detoxification, promoting blood circulation and removing blood stasis	Polydatin	BLM-induced PF rats and A549 cells	Rats (10, 40, 160 mg/kg)	Inhibit TGF-β1/Smad signaling	TGF-β1, Smad2/3, Erk1/2	[156]
						Cell (0–120 μmol/L)			
27					BLM-induced PF rats and HFL-1 cells	Rats (100 mg/kg)	Regulation of TGF-β/Smad signaling	TGF-β, Smad2/3, TNF-α, IL-1β	[157]
						Cell (50, 150 mmol/L)			
28	*Astragali radix*	Fabaceae	Replenishing qi and solidifying the surface, strengthening the upright and dispelling evil	astragaloside IV	A549 cells	Cell (20 mg/mL)	Inhibit the expression of NLRP3	NLRP3, TGF-β1, Smad2/3, α-SMA	[158]
29					BLM-induced PF rats and A549 cells	Rats (20 mg/kg)	Inhibit TGF-β1/PI3K/Akt signaling	TGF-β1, PI3K, Akt	[159]
						Cell (100 μg/mL)			
30	*Tripterygium wilfordii* Hook. f	Ranunculaceae	Clearing heat and detoxification, promoting blood circulation and removing blood stasis	Triptolide	HFL-1 cells	Cell (5, 10, 15, 20 nmol/L)	Regulation of FAK/calin signaling	FAK, calain	[160]
31					paraquat-induced PF mice and BEAS-2B cells	Mice (0.25 mg/kg)	Suppress EMT	TGF-β1, α-SMA, Smad3, E-cadherin	[161]
32	*Schisandrae chinensis* fructus	Magnoliaceae	Astringent and astringent, replenishing qi and invigorating fluid	schisantherin A	BLM-induced PF mice and A549 cells	Mice (1, 2, 4 mg/kg)	Inhibition of ERK signaling	ERK, α-SMA, IL-1β, IL-6, TNF-α	[162]
						Cell (0.625, 10 μmol/L)			
33				schisandrin B	BLM-induced PF mice	Mice (5, 10, 20 mg/kg)	Inhibits Wnt signaling	MMP7, SOD, TGF-β1	[163]
34	Inulae flos	Asteraceae	Clearing heat and detoxification, eliminating phlegm and relieving cough	Inula japonica Thunb.extract	Mlg, CAGA-NIH3T3 cells	Cell (0–80 μmol/L)	Inhibit TGF-β1/Smad3 signaling	TGF-β1, Smad3	[164]
35					BLM-induced PF mice	Mice (100, 200, 400 mg/kg)	Regulates GSK3β signaling	GSK3β, COX-2, GSK3β, p65	[165]
36	Ferulae resina	Apiaceae	Activating qi to relieve pain, warming menstruation and dispelling cold	ferulic acid	Silica-induced PF mice	Mice (100, 300 mg/kg)	Inhibit TGF-β1 signaling	TGF-β1, Smad2/3, CTGF, Slug	[166]
37	*Psoraleae fructus*	Fabaceae	Warm the kidney and consolidate the essence, strengthen the muscles and bones	Psoralen	BLM-induced PF mice and NIH3T3 cells	Mice (5 mg/kg)	Inhibition of ECM precipitate formation	TNF-α, IL‐1β	[167]
						Cell (5, 10, 20, 40 μmol/L)			
38	*Atractylodis rhizoma*	Asteraceae	Invigorating spleen and stomach, tonifying qi and blood	atractylon	OVA-induced asthma mice and TC-1 cells	Mice (25 mg/kg)	Regulate the mmu_circ_0000981/miR-211-5p/ TGFBR2 axis	TFGBR2, Vimentin, α-SMA, collagen	[168]
39	*Angelicae sinensis* radix	Apiaceae	Tonifying blood and activating blood, regulating menstruation and relieving pain	Angelica Sinensis Polysaccharide	BLM-induced PF rats and RLE-6TN cells	Rats (20 mg/kg)	Suppress EMT	DANCR, AUF1	[169]
						Cell (μmol/L)			
40	*Pyrethrum parthenium* (L.) Sm	Asteraceae	Dispelling wind and relieving pain and headache	Parthenolide	BLM-induced PF mice and A549 cells	Mice (12.5, 25, 50 mg/kg)	Inhibits NF-κB/Snail signaling	NF-κB, Snail	[170]
						Cell (5, 10 μmol/L)			
41	Erigeron breviscapus	Asteraceae	Promoting blood circulation and removing blood stasis, clearing heat and detoxification	Scutellarin	BLM-induced PF mice and A549 cells	Mice (30, 60, 90 mg/kg)	Regulation of NF-κB/NLRP3 signaling	NF-κB, NLRP3	[171]
						Cell (0.1, 0.2, 0.4 mmol/L)			
42				Scutellarein	BLM-induced PF mice	Mice (10 mg/kg)	Inhibits PI3K/Akt signaling	PI3K, Akt, Smad2/3, α-SMA	[172]
43	Spirulina platensis			phycocyanin	BLM-induced PF mice	Mice (50 mg/kg)	Regulation of TLR2-MyD88-NF-κB signaling	TLR2, NF-κB	[173]
44	Curcuma aromatica Salisb	Zingiberaceae	Promoting blood circulation and removing blood stasis, reducing swelling and relieving pain	Curdione	BLM-induced PF mice and HPFS cells	Mice (100 mg/kg)	Inhibition of TGF-β/Smad3 signaling	TGF-β1, α-SMA, Collagen 1, Erk1/2	[174]
						Cell (100–500 μmol/L)			
45	Citrus aurantium L	Rutaceae	Soothing the liver and regulating qi, resolving phlegm and relieving cough	Hesperidin	A549 cells	Cell (40–200 μmol/L)	Inhibit TGF-β/Smad2/3 signaling	TGF-β1, Smad2/3, Smad4, Smad7	[175]
46	Alpiniae officinarum rhizoma	Zingiberaceae	Regulating qi and relieving pain, removing dampness and resolving phlegm	Galangin	BLM-induced PF mice and A549 cells	Mice (25, 50 mg/kg)	Suppress EMT	TGF-β1, E-cadherin	[176]
						Cell (0–100 μmol/L)			
47	Aronia melanocarpa	Rosaceae		Cyanidin-3-galactoside	Silica-induced PF mice	Mice (100, 200, 400 mg/kg)	Inhibition of TGF-β/mTOR signaling through the NRF2/HO-1 pathway	TGF-β1, p-mTOR, NRF2	[177]
48	Carthami flos	Asteraceae	Promoting blood circulation and removing blood stasis, regulating menstruation and relieving pain	safflower yellow	paraquat-induced PF rats	Rats (50 mg/kg)	Regulate Hippo signaling	Hippo, Smad2/3, TGF-β1	[178]
49	Juglans mandshurica	Juglandaceae	Moistening the intestines and relieving defecation, tonifying the kidney and strengthening yang	Juglanin	BLM-induced PF mice and MRC-5, MLE-12 cells	mice (80 mg/kg)	Suppress Sting	Sting, MMP-9, α-SMA, TGF-β1	[179]
						Cell (0–160 μmol/L)			
50	Trigonellae semen	Fabaceae	Warming the middle and dispelling cold, regulating qi and relieving pain	Diosgenin	BLM-induced PF rats	Rats (100 mg/kg)	Regulation of TGF-β/Smad signaling	TGF-β1, snail, NF-κB, COX-2, IL-1β	[180]
						Cell (10–30 μmol/L)			
51	Gynostemma pentaphyllum (Thunb.) Makino	Cucurbitaceae	Clearing heat and detoxification, lowering blood pressure	Gypenoside, Gyps	BLM-induced PF mice	Mice (200 mg/kg)	Inhibits AKT/mTOR/c-Myc signaling	AKT, mTOR	[181]
52	Sophorae fla vescentis radix	Fabaceae	Clearing heat and detoxification, diuresis and purging gonorrhea	Matrine	MRC-5 cells	Cell (10 μmol/L)	Inhibit TGF-β/Smad2/3 signaling	TGF-β, Smad2/3	[182]
53	*Vaccinium* spp.	Ericaceae		Pterostilbene	A549 cells	Cell (0–100 μmol/L)	Inhibit TGF-β1 signaling	TGF-β1, Bcl-2, BAX, P62, P-21	[183]
54	Cyanobacteria			C-Phycocyanin	Oleic acid-induced PF mice and A549, HFL-1 cells	Mice (1, 3, 9 mg/kg)	Regulation of TGF-β/Smad and MAPK signaling	TGF-β1, MAPK	[184]
						Cell (10, 30 μmol/L)			
55	*Eclipta prostrata* (L.) L	Saururaceae	Clearing heat and detoxification, stopping bleeding and generating hair	wedelolactone	BLM-induced PF mice and PLFs cells	Mice (2, 10 mg/kg)	Inhibition of RAF1-MAPKs signaling	Col I, α-SAM, AMPK	[185]
						Cell (10 μmol/L)			
56	Nelumbinis semen	Nelumbonaceae	Clear the heart and calm the mind, moisturize the lungs and relieve cough	Lotus Plumule Extract	BLM-induced PF mice	Mice (80, 160, 240 mg/kg)	Inhibit TGF-β1/Smad3 signaling	TGF-β, α-SMA	[186]
57	Gentianae radix et rhizoma	Gentianaceae	Clearing heat, dryness and dampness, purging liver and gallbladder fire	Gentiopicroside	BLM-induced PF mice	Mice (2.5, 10 mg/kg)	Anti-inflammatory	TNF-α, IL‐1β, TGF-β1, CTGF	[187]
58	*Siratia grosvenorii*	Cucurbitaceae	Clear heat and moisturize the lungs and open pharynx	Mogrol	BLM-induced PF mice and NIH3T3 cells	Mice (1, 5, 10 mg/kg)	Regulation of TGF-β1 and AMPK signaling	TGF-β1, AMPK	[188]
						Cell (1, 5, 10 μmol/L)			
59	*Mangifera indica* L.	Anacardiaceae	Clearing heat and detoxification, invigorating the stomach and eliminating food	Mangiferin	BLM-induced PF rats and A549 cells	Rats (40 mg/kg)	Inhibit TGF-β1/Smad2/3 signaling	TLR4, TGF-β1	[189]
						Cell (10 μg/mL)			
60	*Atractylodis rhizoma*	Asteraceae	Invigorate the spleen and appetizer, remove dampness and dissipate phlegm	Atractylodin	BLM-induced PF mice and A549 cells	Mice (50, 100 mg/kg)	Inhibit TGF-β1/Smad signaling	TGF-β1, Snail	[190]
						Cell (0–100 μmol/L)			
61	Indigo naturalis	Brassicaceae	Clearing heat and detoxification, reducing swelling and relieving pain	Indirubin	BLM-induced PF mice and PMLFs HPFs cells	Mice (12.5, 25 mg/kg)	Inhibit TGF-β1/Smad signaling	TGF-β1, Collagen I, α-SMA, Smad2/3	[191]
						Cell (2.5–60 μmol/L)			
62	Ginseng radix et rhizoma	Araliaceae	Replenish qi and nourish blood, invigorate body and quench thirst	ginsenoside Rg3	BLM-induced PF mice and LL 29 cells	Mice (5 mg/kg)	Inhibits nuclear localization of HIF-1α	HIF-1α, TGF-β1	[192]
63	*Inonotus sanghuang*		Clearing heat and detoxification, promoting blood circulation and removing blood stasis	Inonotus sanghuang extract (ISE)	BLM-induced PF mice and A549 cells	Mice (0.6% w/w)	Inhibit TGF-β1/Smad signaling	TGF-β1, Smad2/3	[193]
						Cell (2, 4 μg/mL)			
64	Hippophae fructus	Elaeagnaceae	Invigorate the stomach and eliminate food, relieve cough and expectoration	Isorhamnetin	BLM-induced PF mice	Mice (10, 30 mg/kg)	Suppress EMT	PERK, α-SMA, Collagen I , TGF-β1	[194]
						Cell (25, 50, 100 μmol/L)			
65	Zingiberis rhizoma recens	Zingiberaceae	Warm the middle to dissipate the cold, solve the surface and dissipate the cold	6 gingerol	BLM-induced PF mice and	Mice (100, 250 mg/kg)	Activates SIRT1 signaling signaling	SIRT1, α-SMA, TNF-α, IL-6, IL-1β	[195]
					human embryonic lung fibroblasts MRC-5	Cell (10, 20 μmol/L)			
66	Silybi fructus	Asteraceae	Clearing heat and detoxification, soothing the liver and promoting gallbladder	Silibinin	Silica-induced PF mice	Mice (100, 300 mg/kg)	Anti-inflammatory Inhibits EMT	IL-1β, smad 2/3, α-SMA, TGF-β1	[196]
67	Ampelopsis grossedentata (Hand.-Mazz.) W. T. Wang (Vitaceae)	Lamiaceae	Promoting blood circulation and regulating menstruation, diuresis and detumescence	Dihydromyricetin	BLM-induced PF mice and MLG cells	Mice (50, 100, 200 mg/kg)	Inhibit TGF-β1/Smad signaling	TGF-β1, α-SMA	[197]
68					PMLFs, PHLFs cells	Cell (100, 200, 300 μmol/L)	Regulation of STAT3/p-STAT3/GLUT1 signaling	STAT3,p-STAT3,GLUT1	[198]
69	Dendrobii officinalis caulis	Orchidaceae	Lung heat coughing, deficiency heat dispelling thirst	Polysaccharides from Dendrobium officinale	BLM-induced PF rats	Rats (200 mg/kg)	Inhibition of TGF-β1-Smad2/3 signaling	TGF-β, Smad2/3, α-SMA, Collagen I I	[199]
70	Galla chinensis	Anacardiaceae	Restrain the lungs and reduce the fire, astringent intestines and stop diarrhea	Tannic acid	BLM-induced PF mice	Mice (10 mg/kg)	Inhibit TGF-β receptor signaling	TGF-β, Smad2/3	[200]
						Cell (1, 3 μmol/L)			
71	Camptotheca acuminata Decne	Nyssaceae	Relieve cough and resolve phlegm	Hyperoside	BLM-induced PF mice	Mice (50 mg/kg)	Regulation of AKT/GSK3b signaling	AKT, GSK3b	[201]
72	armeniacae semen amarum	Rosaceae	Moistening the bowels, relieving cough and resolving phlegm	amygdalin	smoking combined with LPS-induced COPD mice and BEAS-2B cells	mice (5, 10, 20 mg/kg)	Inhibit TGF-β/Smad2/3 signaling	TGF-β, Smad2/3	[202]
						Cell (0–2000 μg/mL)			
73	Arenaria kansuensis Maxim	Caryophyllaceae	Clearing heat and detoxification, diuresis and purging gonorrhea	A. kansuensis ethanol extract	paraquat-induced PF rats	Rats (170, 350, 700 mg/kg)	Inhibit NF-κB/TGF-β1/Smad2/3 signal transduction	TGF-β1, Smad2/3	[203]
74	Myrica rubra Sieb	Myricaceae	Moistening the lung and relieving cough, invigorating body and relieving thirst	Myricetin	BLM-induced PF mice and A549, HFL1 cells	Mice (25, 50, 100 mg/kg)	Regulation of TGF-β/Smad and non-Smad signaling	TGF-β, Smad	[204]
						cell (μmol/L)			
75	Epimedii folium	Berberidaceae	Kidney deficiency and impotence, sore waist and knees	Icariin	BLM-induced PF mice and NIH3T3, HLF-1 cells	Mice (0.04, 0.02, 1 mg/kg)	Inhibit TGF-β1 signaling	TGF-β1, α-SMA	[205]
						Cell (3 μmol/L)			
76	Houttuyniae herba	Saururaceae	Heat-clearing and detoxification, sore waist and knees	Sodium Houttuyfonate	BLM-induced PF mice	Mice (45, 90 mg/kg)	Anti-inflammatory	IL-1β, TNF-α	[206]
77	Hypericum longistylum	Hypericaceae	Promoting blood circulation and removing blood stasis, reducing swelling and relieving pain	Hypericum longistylum	MLFC cells	Cell (0–80 μmol/L)	Inhibition of TGF-β/Smad3 signaling	TGF-β	[207]
78	Aurantii fructus immaturus	Rutaceae	Regulating qi stagnation, resolving phlegm and relieving cough	Neohesperidin	BLM-induced PF mice and NIH3T3, MLg, A549 cells	Mice (20 mg/kg)	Inhibition of TGF-β/Smad3 signaling	TGF-β1, Smad2/3, Erk, p-38, JNK	[208]
						cell (0–200 μmol/L)			
79	Multiple plant sources			Hederagenin	BLM-induced PF rats	Rats (10, 20, 50 mg/kg)	Adjust Ras/JNK/NFAT4 axis	JNK, NFAT4	[209]
80	Polyporus	Polyporaceae	Diuresis, detumescence and phlegm	Polyporus Polysaccharide	BLM-induced PF mice and HLF cells	Mice (50, 100 mg/kg)	Inhibit TGF-β1/Smad2/3 signaling	TGF-β1, Smad2/3	[210]
						Cell (1 mg/mL)			
81	Podocarpus nagi	Podocarpaceae	Clearing heat and detoxification, dispelling wind and promoting dampness	Nagilactone D	BLM-induced PF mice and WI-38, VA-13, HFL-1 cells	Mice (1, 3 mg/kg)	Inhibition of TGF-β/Smad3 signaling	TGF-β1, Collagen I, α-SMA	[211]
						Cell (1, 2 μmol/L)			

### Anti-inflammation and anti-oxidation

Oxidative stress is a pathological state in which the body undergoes some kind of stimulation resulting in excessive production of reactive nitrogen radicals and reactive oxygen radicals, leading to an oxidative/antioxidative imbalance. Oxidative stress is a major cellular stressor that can act directly or indirectly on cells, leading to structural necrosis, apoptosis and tissue inflammation [212]. The imbalance between oxidants and antioxidants plays a role in the pathophysiology of IPF, and NADPH oxidase (NOX), which generates reactive oxygen species (ROS), is the primary cause of IPF [213]. Excessive ROS and free radical production can cause lung damage [214]. The level of systemic oxidative stress and disease severity in IPF patients are significantly correlated with dyspnea, as shown by numerous studies [215]. Therefore, anti-oxidative stress is essential for the successful treatment of PF [216]. The inflammatory response is a defense mechanism of the body, and the inflammatory response of the body to different degrees of injury is one of the important factors against lung injury [217, 218]. The pathogenesis of PF may be due to damage to lung epithelial cells by fibrotic stimuli. Therefore, lung inflammation plays an important role in the development of PF. And inflammation is controlled by a variety of cells and cytokines [212, 219] Pro-inflammatory cytokines control tissue differentiation and morphogenesis through adhesion molecules and promote fibrotic responses in lung tissue [220]. Currently, many anti-inflammatory and antioxidant agents have shown effective antifibrotic effects in BLM-induced PF models. Therefore, the imbalance between oxidative stress, oxidants and antioxidants, and inflammation in the development of PF deserve further attention [221, 222].

Emodin is an anthraquinone compound extracted from rhubarb, which has antiviral, anti-cancer, anti-inflammatory and other pharmacological effects [223, 224]. Tian's team found that emodin can significantly reduce the increase of proinflammatory cytokines and oxidative damage caused by BLM [225]. Further experiments found that the anti-inflammatory and antioxidant activities of emodin may be through regulating NF-κB and Nrf2 signal pathways. This study preliminarily revealed the anti-inflammatory and antioxidant activities of emodin in improving PF, and preliminarily explored the possible signal pathways involved. However, the mechanism by which emodin exerts its biological activity still needs to be further explored in order to better play its potential in the treatment of PF. Qi's team found the protective effect and potential mechanism of chrysophanol in IPF through research [226]. The research results shown that chrysophanol can effectively reduce ECM deposition and inflammatory cytokine levels in PF model mice, and chrysophanol can also inhibit Wnt/β-catenin signaling pathway and inhibition of lung fibroblast proliferation to alleviate BLM induced mouse PF. This study demonstrates that chrysophanol has anti-inflammatory biological activity, but further experimental verification is needed. In addition to emodin and chrysophanol, rhubarb also contains many active ingredients with anti-inflammatory and antioxidant effects. The improvement of PF by these active ingredients deserves further research.

Studies have shown that ethyl acetate extract of Salvia miltiorrhiza can reduce the degree of active oxygen-related PF by targeting Nrf2-NOX4 REDOX equilibrium [227, 238]. Tanshinone IIA is a bioactive ingredient extracted from Salvia miltiorrhiza with anti-inflammatory, antioxidant and anti-fibrotic properties. Feng's research team studied the protective effect of Tanshinone IIA on silica-induced PF and its potential mechanism [229], and the results showed that tanshinone IIA could down-regulate the level of oxidative stress markers in silicosis rats and attenuate pulmonary inflammatory response. In addition, Tanshinone IIA may protect the lung from silica damage by inhibiting TGF-β1/Smad signaling, inhibiting NOX4 expression, and activating the Nrf2/ARE pathway. An’s team found a similar phenomenon in mice with bleomycin-induced PF treated with tanshinone IIA [152]. The study found that tanshinone IIA can inhibit PF by activating Nrf2, regulating REDOX homeostasis and glutamine breakdown. Liu's study showed that salvianolic acid B can reduce experimental lung inflammation by protecting endothelial cells from oxidative stress [230], further demonstrating that the anti-inflammatory effects of Salvianolic acid B may be mediated through MAPK and NF-κB signaling pathways. Notably, Salvianolic acid B and Tanshinone IIA sulfonates reduce PF by affecting the inflammatory system and controlling the TGF-β1 pathway, which may be the result of a synergistic effect between the two drugs [153]. In summary, many active compounds in salvia miltiorrhiza have pharmacological effects on improving PF, and the synergistic effect between these compounds is worthy of further study. In addition to the above-mentioned TCM that can improve PF, the detailed information of other TCM studies in the past five years that can improve PF through anti-inflammation and anti-oxidation were summarized and presented in Table 2.

**Table 2:** Details about some traditional Chinese medicines improving pulmonary fibrosis by anti-inflammation and anti-oxidation effects

No	Source	Genus information	Traditional efficacy	Active compound	Experiment model	Administration dose	Therapeutic target	Cytokine	Refs.
1	Salviae miltiorrhizae radix et rhizoma	Lamiaceae	Promoting blood circulation and removing blood stasis, reducing swelling and relieving pain	Salvia miltiorrhiza	BLM-induced PF mice and NIH3T3 cells	Mice (21, 40, 80 mg/kg)	Anti-oxidation	Nrf2, NOX4	[228]
						Cell (0.1, 1, 3 μmol/L)			
2				Tanshinone IIA	Silica-induced PF rats	Rats (25 mg/kg)	Regulation of TGF-β1/Smad and Nrf2/ARE signaling	TGF-β1, Nrf2	[229]
3					BLM-induced PF mice and PLFs, NIH-3T3 cells	Mice (5, 10, 20 mg/kg)	Inhibition of TGF-β1-Smad3 signaling	Nrf2, TGF-β1	[152]
						Cell (0.01–500 μmol/L)			
4				Sodium Tanshinone IIA sulfonate	Silica-induced PF rats and A549, RLE-6TN, MRC-5, NIH-3T3 cells	Rats (25 mg/kg)	Up-regulation of Nrf2 nuclear expression	Nrf2, Trx, TrxR	[231]
						Cell (μmol/L)			
5				Salvianolic acid B	BLM-induced PF mice and hy926 cells	Mice (10 mg/kg)	Anti-inflammatory and antioxidant	IL-1β, TNF-α, NF-κB	[230]
						Cell (50 μg/mL)			
6					BLM-induced PF rats and MRC-5 cells	Rats (20 mg/kg)	Regulates Nrf2 signaling	Nrf2, α-SMA, TGF-β1	[138]
						Cell (40 μmol/L)			
7				Salvianolic acid B/sodium tanshinone IIA sulfonate	THP-1 cells	Cell (0–600 μg/mL)	Inhibit TGF-β1 signaling	TGF-β1, α-SMA	[153]
8	Curcumae longae rhizoma	Zingiberaceae	Promoting blood circulation and removing blood stasis, Regulating menstruation and relieving pain	Curcumin	BLM-induced PF mice	Mice (75 mg/kg)	Inhibit NF-κB signaling	AMPK, COX-2	[232]
9					paraquat-induced PF rats	Rats (200 mg/kg)	Improve pulmonary fibrosis	Smad 4, Smurf 2	[233]
10					LMSCs cells	Cell (2.5, 5, 10, 20 μmol/L)	Regulation of Akt/Nrf2/HO-1 signaling	Akt, Nrf2, HO-1	[234]
11					BLM-induced PF rats	Rats (300 mg/kg)	Inhibition of ECM precipitate formation	/	[235]
12					A549 cells	Cell (20 μmol/L)	Inhibit TGF-β1/Smad/non Smad signaling	TGF-β1, Smad	[147]
13	Rhei radix et rhizoma	Polygonaceae	Purging and defecating, clearing heat and detoxification	Chrysophanol	BLM-induced PF mice	Mice (10 mg/kg)	Inhibits Wnt/β-catenin signaling	β-catenin, Bax, TNF-α, IL-1β	[226]
14				Emodin	BLM-induced PF rats and A549 cells	Rats (20 mg/kg)	Anti-inflammatory and antioxidant	IL-1β, IL-6, TNF-α, NF-κB	[225]
						Cell (60 μmol/L)			
15				Rhapontin	BLM-induced PF rats and THP-1 cells	Rats (25, 50, 100 mg/kg)	Regulation of TGF-β/Smad signaling	TGF-β1, Smad	[137]
						Cell (50 μmol/L)			
16	Polygoni cuspidati rhizoma et radix	Polygonaceae	Clearing heat and detoxification, promoting blood circulation and removing blood stasis	Polydatin	MTX‐induced PF rats	Rats (25, 50, 100 mg/kg)	Inhibit TGF-β1 signaling	TGF-β1, HYP, α-SMA, TNF-α	[236]
17					BLM-induced PF rats and A549 cells	Rats (10, 40, 160 mg/kg)	Inhibit TGF-β1/Smad signaling	TGF-β1, Smad2/3, Erk1/2	[156]
						Cell (0–120 μmol/L)			
18					BLM-induced PF rats and HFL-1 cells	Rats (100 mg/kg)	Regulation of TGF-β/Smad signaling	TGF-β, Smad2/3, TNF-α, IL-1β	[157]
						Cell (50, 150 mmol/L)			
19	Astragali radix	Fabaceae	Replenishing qi and solidifying the surface, strengthening the upright and dispelling evil	Baicalin	BLM-induced PF rats	Rats (25, 100 mg/kg)	Activate SOD	/	[237]
20				Astragaloside IV	Silica-induced PF rats	Rats (20 mg/kg)	Inhibit TGF-β1/Smad signaling	TGF-β1, α-SMA	[238]
21					A549 cells	Cell (20 mg/mL)	Inhibit the expression of NLRP3	NLRP3, TGF-β1, Smad2/3, α-SMA	[158]
22	Tripterygium wilfordii Hook. f	Ranunculaceae	Clearing heat and detoxification, promoting blood circulation and removing blood stasis	Triptolide	radiation-induced PF mice and NIH3T3 cells	Mice (0.25 mg/kg)	Inhibit NF-κB signaling	NF-κB, LOX, IκBα	[239]
						cell (5 ng/mL)			
23					HFL-1 cells	Cell (5, 10, 15, 20 nmol/L)	Regulation of FAK/calin signaling	FAK, calain	[160]
24				Isorhynchophylline	Silica-induced PF mice	Mice (20 mg/kg)	Anti-inflammatory	/	[240]
25	Andrographis herba	Acanthaceae	Clearing heat and detoxification, cooling blood	Andrographolide	BLM-induced PF rats and A549 cells	Rats (10 mg/kg)	Regulation of AKT/mTOR signaling	AKT, mTOR	[134]
26					A549 cells	Cell (0–20 μmol/L)	Inhibits A and B signaling	TGF-β1, Smad2/3, MMP-9	[133]
27	Schisandrae chinensis fructus	Magnoliaceae	Astringent and astringent, replenishing qi and invigorating fluid	Schisantherin A	BLM-induced PF mice and A549 cells	Mice (1, 2, 4 mg/kg)	Inhibition of ERK signaling	ERK, α-SMA, IL-1β, IL-6, TNF-α	[162]
						Cell (0.625, 10 μmol/L)			
28				Schisandrin B	BLM-induced PF mice	Mice (5, 10, 20 mg/kg)	Inhibits Wnt signaling	MMP7, SOD, TGF-β1	[163]
29	glycyrrhizae radix et rhizoma	Fabaceae	Nourishes qi and nourishes yin, clears away heat and detoxifies	Licorice extract	paraquat-induced PF mice and A549, HepG2 cells	Mice (20, 40, 60 mg/kg)	Regulates Nrf2 signaling	Nrf2, TGF-β1, CYP3A4, MDA	[241]
						Cell (0–100 μmol/L)			
30				Deglycyrrhizinated licorice	BLM-induced PF rats	Rats (75, 150, 300 mg/kg)	Anti-inflammatory and antioxidant	/	[242]
31	Ferulae resina	Apiaceae	Activating qi to relieve pain, warming menstruation and dispelling cold	Ferulic acid	Silica-induced PF mice	Mice (100, 300 mg/kg)	Inhibit TGF-β1 signaling	TGF-β1, Smad2/3, CTGF, Slug	[166]
32	Birch bark	Betulaceae	Clear away heat and dampness, detoxify	Betulinic acid	BLM-induced PF mice and MLG, PPF cells	Mice (20, 40, 80 mg/kg)	Inhibit Wnt/β-catenin signaling	β-catenin, Col 1, α-SMA	[69]
						Cell (5, 10, 20 μmol/L)			
33	Bletillae rhizoma	Orchidaceae	Convergence to stop bleeding, reduce swelling and promote muscle growth	Bletilla striata	RAW264.7 cells	Cell (2.5 μg/mL)	Anti-inflammatory	/	[243]
34	Chelidonii herba	Papaveraceae	Clearing heat and detoxification, reducing swelling and relieving pain	Chelerythrine	BLM-induced PF mice	Mice (0.375, 0.75 mg/kg)	activate Nrf2/ARE signal transduction	Nrf2, ARE	[244]
35	Stemonae radix	Stemonaceae	Expelling phlegm and relieving cough, killing insects and expelling Ascaris	Stemona alkaloids	BLM-induced PF mice and PFB cells	Mice (30, 60 mg/kg)	Inhibits JAK2/STAT3 and CXCR4/PI3K/AKT1 signaling	STAT3, PI3K, AKT1	[245]
						Cell (1, 10, 100 μg/mL)			
36	Atractylodis rhizoma	Asteraceae	Invigorate the spleen and appetizer, remove dampness and dissipate phlegm	Atractylenolide III	BLM-induced PF rats	Rats (0.6, 1.2, 2.4 mg/kg)	activate Nrf2/NQO1/HO-1 signal transduction	Nrf2, NQO1, HO-1	[246]
37	Rehmanniae radix	Oleaceae	Nourishing yin and tonifying blood, clearing heat and cooling blood	catalpol	BLM-induced PF rats	Rats (10, 20, 40 mg/kg)	Antioxidant inhibits EMT	TGF-β, Smad3	[247]
38	Erigeron breviscapus	Asteraceae	Promoting blood circulation and removing blood stasis, clearing heat and detoxification	Scutellarin	BLM-induced PF mice and A549 cells	Mice (30, 60, 90 mg/kg)	Regulation of NF-κB/NLRP3 signaling	NF-κB, NLRP3	[171]
						cell (0.1, 0.2, 0.4 mmol/L)			
39	Spirulina			Phycocyanin	BLM-induced PF mice	Mice (50 mg/kg)	Anti-inflammatory	IL-6, TNF-α	[248]
40	Various plant sources			Epicatechin	BLM-induced PF mice	Mice (20, 50, 100 mg/kg)	Anti-inflammatory and antioxidant	/	[249]
41				Sinapic acid	BLM-induced PF rats	Rats (10, 20 mg/kg)	Inhibit NF-κB/NRF2/HO-1 signaling	NF-κB, NRF2, HO-1	[250]
42				Pinocembrin	BLM-induced PF mice	Mice (5, 25, 50 mg/kg)	Inhibit TLR4/NF-κB/NLRP3 signaling	NF-κB, NLRP3	[251]
						cell (100, 200, 300 μmol/L)			
43	Cymbopogon winterianus	Cymbopogon		Essential oil of Cymbopogon winterianus	BLM-induced PF rats	Rats (50, 100, 200 mg/kg)	Inhibit TGF-β1 signaling	TGF-β1, SOD. MDA	[252]
44	Citrus fruits	Rutaceae	Invigorate the spleen and replenish qi, moisturize the lungs and relieve cough	Hesperidin	BLM-induced PF rats	Rats (25, 50, 100 mg/kg)	Inhibits TGF-β1/Smad3/AMPK and I-κBα/NF-κB signaling	TGF-β1, NF-κB	[253]
45	Alpiniae officinarum rhizoma	Zingiberaceae	Regulating qi and relieving pain, removing dampness and resolving phlegm	Galangin	BLM-induced PF mice and A549 cells	Mice (25, 50 mg/kg)	Suppress EMT	E-cadherin, vimentin, α-SMA, MMP-9	[176]
						Cell (0–100 μmol/L)			
46	Puerariae lobatae radix	Fabaceae	Relieving cold, sweating and detoxification	Puerarin	HLF1 cells	Cell (200, 400, 600 μg/mL)	Inhibition of TGF-β/Smad3 signaling	TGF-β1, Caspase3, Bcl-2, Smad3	[254]
47	Glycyrrhizae radix et rhizoma	Fabaceae	Clearing heat and detoxification, moistening the lungs and relieving cough	Glycyrrhiza glabra	BLM-induced PF rats	Rats (500 mg/kg)	Anti-inflammatory and antioxidant	HYP, LPO	[255]
48	Laminaria japonica	Phaeophyta	Clearing heat and detoxification, softening and dispersing knots	Low molecular weight fucoidan	BLM-induced PF mice and A549 cells	Mice (25, 50, 100 mg/kg)	Antioxidant inhibits fibrosis	NRF-2, HO-1, NQO1	[256]
						Cell (50, 100, 200 μg/mL)			
49	Carthami flos	Asteraceae	Promoting blood circulation and removing blood stasis, regulating menstruation and relieving pain	Hydroxysafflor Yellow A	MRC-5 cells	Cell (5, 15, 45 μmol/L)	Inhibit NF-κB/AP-1 signaling	NF-κB, AP-1	[257]
50					BLM-induced PF rats	Rats (35.6, 53.3, 80 mg/kg)	Anti-inflammatory	α-SMA, IL-1β, IL-6, TNF-α, TGF-β	[258]
51	Rhodiolae crenulatae radix et rhizoma	Crassulaceae	Replenishing qi and activating blood circulation, dredging pulse and relieving asthma	Rutin	BLM-induced PF rats	Rats (50, 100 mg/kg)	Regulation of TGF-β1/α-SMA/Col I/III signaling	TGF-β1, α-SMA	[259]
52	Juglans	Juglandaceae	Moistening the bowels, relieving cough and resolving phlegm	Juglanin	BLM-induced PF mice and MRC-5, MLE-12 cells	Mice (80 mg/kg)	Suppress sting	Sting, MMP-9, α-SMA, TGF-β1	[179]
						Cell (0–160 μmol/L)			
53	Trigonellae semen	Fabaceae	Warming the middle and dispelling cold, regulating qi and relieving pain	Diosgenin	BLM-induced PF rats	Rats (100 mg/kg)	Regulation of TGF-β/Smad signaling	TGF-β1, snail, NF-κB, COX-2, IL-1β	[180]
						Cell (10–30 μmol/L)			
54	Coptidis rhizoma	Ranunculaceae	Clearing heat and dryness, purging fire and detoxification	Berberine	BLM-induced PF mice	Mice (50, 100, 200 mg/kg)	Activate PPAR-γ	HGF, PPAR-γ	[260]
55	Astragali radix/ferulae resina	Fabaceae/Apiaceae	Replenishing qi and solidifying the surface, strengthening the upright and dispelling evil	Astragaloside IV/ferulic acid	BLM-induced PF mice	Mice (24 + 40.8 mg/kg)	Inhibit TGF-β1/Smad3 signaling	TGF-β1, Nrf2	[261]
56	Scutellariae radix	Scutellaria	Clearing away heat and dampness, purging fire and detoxifying	Baicalin	BLM-induced PF rats and RPF	Rats (50 mg/kg)	Regulation of CaMKII and PI3K/AKT signaling	PI3K, AKT	[141]
						Cell (20, 40, 60, 80 μg/mL)			
57				Calycosin, CA	BLM-induced PF mice and MLE-12 cells	Mice (7, 14 mg/kg)	Inhibit AKT/GSK3β/β-catenin signaling	AKT, GSK3β	[144]
						cell (0–80 μmol/L)			
58	Centellae herba	Apiaceae	Clearing heat and detoxification, promoting diuresis and detumescence	Asiaticoside	BLM-induced PF mice	Mice (50 mg/kg)	Activates cAMP and RAP1 signaling	A2AR, RAP1	[262]
59					BLM-induced PF mice	Mice (50 mg/kg)	Up-regulation of BMP7/Smad1/5 signaling	BMP7, Smad1/5	[263]
60	Tribuli fructus	Cucurbitaceae	Soothing the liver and relieving depression, promoting diuresis and reducing swelling	Terrestrosin D	BLM-induced PF mice	Mice (10 mg/kg)	Anti-inflammatory	HYP, IL-6, IL-8, TGF-β, PDGF-AB	[264]
61	Zingiberis rhizoma recens	Zingiberaceae	Warm the middle to dissipate the cold, solve the surface and dissipate the cold	Zingerone	BLM-induced PF rats	Rats (50, 100 mg/kg)	Affects TGF-β1 and iNOS signaling	TGF-β1, MDA, SOD, TNF-α, IL-1β	[265]
62	Lonicerae japonicae caulis	Caprifoliaceae	Clearing heat and detoxification, relieving surface and dissipating heat	Blue honeysuckle extract	Silica-induced PF mice and HLN cells	Mice (100, 200, 400 mg/kg)	Regulation of NRF2/HO-1 MAPK signaling	NRF2, HO-1, MAPK	[266]
63				Cyanidin-3-glucoside	Silica-induced PF mice	Mice (100, 200, 400 mg/kg)	Inhibits STAT1/3 signaling	STAT1, STAT3	[267]
64	Blueberry	Ericaceae		Pterostilbene	LPS-induced PF mice	Mice (12.5, 25, 50 mg/kg)	Activation of Keap-1/Nrf2 inhibits A20/NF-κB and NLRP3 signaling	NF-κB, NLRP3	[268]
65	Cyanobacteria			C-Phycocyanin	Oleic acid-induced PF mice and A549, HFL-1 cells	Mice (1, 3, 9 mg/kg)	Regulation of TGF-β/Smad and MAPK signaling	TGF-β1, MAPK	[184]
						Cell (10, 30 μmol/L)			
66	Eclipta prostrata L	Asteraceae	Heat-clearing and detoxification, black hair	Wedelolactone	BLM-induced PF mice and PLFs cells	Mice (2, 10 mg/kg)	Inhibition of RAF1-MAPKs signaling	Col I, α-SAM, AMPK	[185]
						cell (10 μmol/L)			
67	Nelumbinis semen	Nelumbonaceae	Clear the heart and calm the mind, moisturize the lungs and relieve cough	Lotus plumule extract	BLM-induced PF mice	mice (80, 160, 240 mg/kg)	Inhibit TGF-β1/Smad3 signaling	TGF-β1, α-SMA	[186]
68	Oxytropis falcata Bunge	Fabaceae	Clearing heat and detoxification, dispelling wind and dispersing blood stasis	Flavonoids of Oxytropis falcata	BLM-induced PF rats	Rats (100, 200, 400 mg/kg)	Regulates JAK/STAT1 signaling	SOCS, p-JAK1	[269]
69	gentianae radix et rhizoma	Gentianaceae	Clearing heat, dryness and dampness, purging liver and gallbladder fire	Gentiopicroside	BLM-induced PF mice	Mice (2.5, 10 mg/kg)	Anti-inflammatory	TNF-α, IL‐1β, TGF-β1, CTGF	[187]
70	Cervi cornu pantotrichum		Tonifying the kidney and tonifying essence, strengthening muscles and bones	Pilose antler peptide	BLM-induced PF mice and A549 cells	Mice (50, 100 mg/kg)	Regulation of ROCK/NF-κB signaling	NF-κB, MPO, SOD, IL-1β, IL-6, TNF-α, IκBα	[270]
71	Siratia grosvenorii	Cucurbitaceae	Clear heat and moisturize the lungs, invigorate body and quench thirst	Mogrol	BLM-induced PF mice and NIH3T3 cells	Mice (1, 5, 10 mg/kg)	Regulation of TGF-β1 and AMPK signaling	TGF-β1, AMPK	[188]
						Cell (1, 5, 10 μmol/L)			
72	Gallnut	Anacardiaceae	Stop diarrhea, converge, astringent intestines	Gallic acid derivative	BLM-induced PF mice	Mice (75, 150, 300 mg/kg)	Anti-inflammatory and antioxidant	NOX4, Nrf2	[271]
73	Ophiopogonis radix	Liliaceae	Nourish yin and moisturize dryness, clear the heart and calm the mind	Ophiopogonin C	radiation-induced PF mice	Mice (3 mg/kg)	Inhibit TGF-β1 signaling	TGF-β1, IL-6, HYP, SOD, MDA, MMP-2	[272]
74	Mangifera indica	Anacardiaceae	Clearing heat and detoxification, invigorating the stomach and eliminating food	Mangiferin	BLM-induced PF rats and A549 cells	Rats (40 mg/kg)	Inhibit TGF-β1/Smad2/3 signaling	TLR4, TGF-β1	[189]
						Cell (10 μg/mL)			
75	Rosmarinus officinalis	Lamiaceae		Rosmarinic Acid	radiation-induced lung damage rats	Rats (30, 60, 120 mg/kg)	Inhibits RhoA/Rock signaling	RhoA, Rock	[273]
76				Carnosol	BLM-induced PF rats	Rats (10, 20, 40 mg/kg)	Anti-inflammatory and antioxidant	HYP, MDA, PC, NO, GSH, SOD, TNF-α, IL-6	[274]
77	Oroxyli semen	Palmaceae	Detoxify and remove phlegm, relieve cough and asthma	Chrysin	BLM-induced PF rats	Rats (50 mg/kg)	Inhibit TGF-β1 signaling	TGF-β1, TXNIP	[275]
78	Aucklandiae radix	Asteraceae	Regulate spleen and stomach, remove dampness and eliminate phlegm	Costunolide	BLM-induced PF mice and HELF cells	Mice (10, 20 mg/kg)	Regulation of NF-kB and TGF-β1/Smad2/Nrf2-NOX4 signaling	NF-kB, TGF-β1	[276]
						Cell (10, 20 μmol/L)			
79	Grape			Resveratrol	BLM-induced PF rats and BEAS-2B cells	Rats (25, 50, 100 mg/kg)	Inhibits HIF-1α and NF-κB signaling	HIF-1α, NF-κB	[277]
						Cell (2.5, 5, 10 mg/mL)			
80					PM-induced PF mice and BEAS-2B cells	Mice (100 mg/kg)	Inhibit the expression of NLRP3	NLRP3, TGF-β1, IL-1β, IL-6, TNF-α, α-SMA	[278]
						Cell (1, 5 μmol/L)			
81					FCA-induced arthritis rats	Rats (10 mg/kg)	Regulation of JAK/STAT/RANKL signaling	JAK, STAT	[279]
82				Grape seed proanthocyanidin extract (GSPE),	BLM-induced PF mice and A549 cells	Mice (30, 60, 90; 50, 100, 150 mg/kg)	Inhibition of oxidative stress inhibits epithelial cell apoptosis	HYP, TNF-α, IL-1β, IL-6, PARP, Bak	[280]
						Cell (1 μg/mL)			
83	Indigo naturalis	Brassicaceae	Clearing heat and detoxification, reducing swelling and relieving pain	Indirubin	BLM-induced PF mice and PMLFs HPFs cells	Mice (12.5, 25 mg/kg)	Inhibit TGF-β1/Smad signaling	TGF-β1, ALT, CR, HYP, Collagen I, α-SMA	[191]
						Cell (2.5–60 μmol/L)			
84	*Artemisia annua* L.	Asteraceae	Clear deficiency heat and remove bone steaming	Dihydroartemisinin	BLM-induced PF mice	Mice (25, 50, 100 mg/kg)	Reduced expression of TNF-α, IL-6 and TGF-β1 via TGF-β/JAK2/STAT3 signaling	TGF-β1, JAK2, STAT3	[281]
85					BLM-induced PF rats	Rats (50 mg/kg)	anti-oxidation	SOD, GSH, MDA, HO-1, Nrf2	[282]
86	Nervilia fordii	Orchidaceae	Clearing heat and moistening the lungs, relieving cough and resolving phlegm	The extract of Nervilia fordii	BLM-induced PF rats and CT6 cells	Rats (100, 200, 400 mg/kg)	Inhibit TGF-β1/Smad signaling	TGF-β1, HYP, MPO, T-AOC, GSH, SOD, TNF-α	[283]
						Cell (100, 200, 500 μg/mL)			
87	Cinnamomi cortex	Lauraceae	Warming the meridians and dispelling cold, dredging yang and reaching the camp	Trans-cinnamaldehyde	V79-4 cells	Cell (0–50 μmol/L)	Activates Nrf2/HO-1 signaling	Nrf2, HO-1, ROS, MMP	[284]
88	Notoginseng radix et rhizoma	Araliaceae	Promoting blood circulation and removing blood stasis, clearing heat and detoxification	Panax notoginseng saponin	ferric trichloride-induced PF Japanese rabbits	Rabbit(50 mg/kg)	Alleviate lung damage	IL-6, NF-κB	[285]
89	Inonotus sanghuang		Clearing heat and detoxification, promoting blood circulation and removing blood stasis	Inonotus sanghuang extract (ISE)	BLM-induced PF mice and A549 cells	Mice (0.6% w/w)	Inhibit TGF-β1/Smad signaling	TGF-β1, Smad2/3	[193]
						Cell (2, 4 μg/mL)			
90	Kaempferiae rhizoma	Zingiberaceae	Promoting qi to relieve pain and regulating qi in a broad way	Alpha-Mangostin	BLM-induced PF mice	Mice (10 mg/kg)	Regulates AMPK signaling	AMPK, α-SMA, Smad2/3, Col I, MMP-9	[286]
91	Cnidii fructus	Loranthaceae	Warming the kidney to help yang, tonifying essence and astringent	Osthole	BLM-induced PF mice	Mice (40 mg/kg)	Downregulation of TGF-β1/nox4 signaling	TGF-β1, nox4	[287]
92	Zingiberis rhizoma recens	Zingiberaceae	Warm the middle to dissipate the cold, solve the surface and dissipate the cold	6 gingerol	BLM-induced PF mice and	Mice (100, 250 mg/kg)	Activates SIRT1 signaling signaling	SIRT1, α-SMA, TNF-α, IL-6, IL-1β	[195]
					human embryonic lung fibroblasts MRC-5	Cell (10, 20 μmol/L)			
93	Amaryllidaceae	Amaryllidaceae	Dispel wind and detoxification, kill insects and stop itching	Lycorine	BLM-induced PF mice and BMDMs cells	Mice (5, 10, 20 mg/kg)	Inhibit the expression of NLRP3	NLRP3, MPO, IL-6, IL-1β, α-SMA	[288]
						Cell (0–40 mmol/L)			
94	Salvia officinalis	Lamiaceae	Clearing heat and detoxification, dispelling wind and relieving pain	Salvia officinalis	BLM-induced PF rats	Rats (50, 100, 150 mg/kg)	Anti-oxidation	CAT, MDA, SOD	[289]
95	Silybi fructus	Asteraceae	Clearing heat and detoxification, soothing the liver and promoting gallbladder	Silibinin	Silica-induced PF mice	Mice (100, 300 mg/kg)	Anti-inflammatory Inhibits EMT	MDA, GSH, HYP, IL-6, IL-1β, IL-17, TGF-β1	[196]
96	Ampelopsis grossedentata (Hand.-Mazz.) W. T. Wang (Vitaceae)	Lamiaceae	Promoting blood circulation and regulating menstruation, diuresis and detumescence	Dihydromyricetin	BLM-induced PF mice and MLG cells	Mice (50, 100, 200 mg/kg)	Inhibit TGF-β1/Smad signaling	TGF-β1, α-SMA	[197]
97	Schisandrae chinensis fructus/glycyrrhizae radix et rhizoma	Magnoliaceae/Fabaceae	Astringent and astringent, replenishing qi and invigorating fluid	Schizandrin B + Glycyrrhizic acid	BLM-induced PF mice	Mice (100, 75, 100 + 75 mg/kg)	Regulation of TGF-β/Smad2 signaling	NOX1, Smad2	[290]
98	Croci stigma	Iridaceae	Promoting blood circulation and removing blood stasis, regulating menstruation and relieving pain	Crocin	BLM-induced PF rats	Rats (25 mg/kg)	Anti-inflammatory and antioxidant	HYP, GSH, CAT, SOD, TNF-α, MDA	[291]
99					BLM-induced PF rats	Rats (20 mg/kg)	Regulation of NRF2 and HO-1 signaling	NRF2, HO-1	[292]
100	Rhododendron brachycarpum G. Don	Ericaceae	Clearing heat and detoxification, reducing swelling and relieving pain	Hyperoside	BLM-induced PF mice	Mice (50 mg/kg)	Regulation of AKT/GSK3b signaling	AKT, GSK3b	[203]
101	Arenaria kansuensis	Caryophyllaceae	Clearing heat and detoxification, diuresis and purging gonorrhea	A. kansuensis ethanol extract	paraquat-induced PF rats	Rats (170, 350, 700 mg/kg)	Inhibit NF-κB/TGF-β1/Smad2/3 signal transduction	NF-κB, TGF-β1, Smad2/3	[203]
102	Morchella esculenta	Morchellaceae	Tonifying the kidney, tonifying qi, nourishing blood and calming the mind	FMP-1	A549 cells	Cell (50–300 μg/mL)	Regulation of PI3K/AKT-Nrf2/HO-1 signaling	ROS, PI3K	[293]
103	Leonuri herba	Lamiaceae	Promoting blood circulation and regulating menstruation, reducing swelling and relieving pain	Leonurine	BLM-induced PF mice	Mice (50, 100 mg/kg)	Up-regulation of AKT signaling	AKT, ECAD, TGF-β, BAX, ACTA2	[294]
104	Epimedii folium	Berberidaceae	Kidney deficiency and impotence, sore waist and knees	Icariin	BLM-induced PF rats	Rats (60 mg/kg)	Inhibit hippo signaling	YAP, IL-1β, IL-6, TGF-β1, TNF-α	[295]
105	Houttuyniae herba	Saururaceae	Heat-clearing and detoxification, sore waist and knees	Sodium Houttuyfonate	BLM-induced PF mice	Mice (45, 90 mg/kg)	Anti-inflammatory	IL-1β, TNF-α	[206]
106	Anemarrhenae rhizoma	Liliaceae	Clearing heat and moistening dryness, promoting fluid to quench thirst	total extract of Anemarrhenae Rhizoma (TEAR)	BLM-induced PF rats	Rats (1.33, 4, 12 g/kg)	Inhibit TGF-β1/Smad signaling	TGF-β1, HYP, COlI, ColIII, MPO, NO	[296]
107	Hedara nepalensis var.sinensis	Araliaceae	Clearing heat and detoxification, dispelling wind and promoting dampness	Hederagenin	BLM-induced PF rats	Rats (10, 20, 50 mg/kg)	Adjust Ras/JNK/NFAT4 axis	JNK, NFAT4	[209]

### Improve extracellular matrix deposition

ECM is a three-dimensional network of non-cellular macromolecules, made primarily of collagen, non-collagenous proteins, and glycoproteins, among others [297]. A pathological characteristic of PF is the massive buildup of ECM in the interstitium. Excessive ECM deposition promotes alveolar structural loss, which generates or aggravates PF. Initial damage to alveolar epithelial cells stimulates the production of multiple pro-fibrotic cytokines that induce fibroblast proliferation, aggregation, and transdifferentiation, generating myofibroblasts that secrete stronger ECM, releasing high levels of ECM, and promoting the development of PF [298]. Under pathological situations, excessive deposition of ECM in the interstitial lung can stimulate ECM synthesis and alter the regulatory function of matrix metalloproteinases (MMPs)/TIMPs [299, 300]. Under pathological situations, excessive deposition of ECM in the interstitial lung can stimulate ECM synthesis and alter the regulatory function of matrix metalloproteinases (MMPs)/TIMPs [301]. The majority of animal investigations have concentrated on the independent detection of TGF-β1, Smads, and MMPs/TIMPs, and the precise mechanism of action is still unknown. Furthermore, Smads, MMPs, and TIMPs comprise several family members whose activities and mutual effects have yet to be thoroughly investigated [45].

In addition to its anti-inflammatory and antioxidant properties, Salvia miltiorrhiza can also play an anti PF role by inhibiting ECM deposition. Feng’s research suggests that tanshinone IIA inhibits TGF-β1/Smad signaling pathway to reduce silica induced PF [302]. Further research found that tanshinone IIA may improve silicosis fibrosis by inhibiting the EMT phase. Cryptotanshinone is a lipophilicity compound derived from salvia miltiorrhiza, which has antioxidant, anti-inflammatory and anti-angiogenesis properties [303]. Zhang research team found that cryptotanshinone improved the lung function of the rat model of bleomycin induced PF, alleviated pathological changes and inhibited ECM precipitation [304]. This experiment found that cryptotanshinone may prevent PF by inhibiting Smad and STAT3 signaling pathways through cell experiments. In conclusion, many active compounds in Salvia miltiorrhiza have good anti PF effects in terms of anti inflammation, anti-oxidation, inhibition of EMT and inhibition of ECM precipitation.

Astragalus membranaceus is a TCM with various pharmacological effects such as enhancing immune function, protecting lung function, and reducing oxidative stress. Astragaloside IV is one of the most important active ingredients in astragalus membranaceus. Some studies have shown that astragaloside IV not only improves the secretion of collagen induced by bleomycin, but also reduces the level of type III collagen, serum laminin and hyaluronic acid in lung homogenate [305]. These findings indicated that astragaloside IV can effectively inhibit ECM deposition in PF mice, providing experimental data support for its use as a candidate compound for the treatment of PF. Li’s team also studied the anti PF effect of astragaloside IV [306]. The difference is that this experiment uses the silicon induced PF model. The experiment shown that astragaloside IV can effectively inhibit the formation of silicosis fibrosis. Further cell experiments have found that astragaloside IV can inhibit ECM precipitation in fibroblasts, and its mechanism of action may be related to TGF-β1/Smad signaling pathway is involved. These results suggest that astragalus may have the potential to improve PF by inhibiting ECM precipitation in a variety of disease induced PF. In addition to the above-mentioned TCM that can improve PF, the detailed information of other TCM studies in the past five years that can improve PF through inhibition of ECM deposition were summarized and presented in Table 3.

**Table 3:** Details about some traditional Chinese medicines improving pulmonary fibrosis by inhibiting extracellular matrix deposition

No	Source	Genus information	Traditional efficacy	Active compound	Experiment model	Administration dose	Therapeutic target	Cytokine	Refs.
1	Andrographis herba	Acanthaceae	Clearing heat and detoxification, cooling blood	Andrographolide	NIH 3T3, PLF cells	Rats (10 mg/kg)	Inhibits TGF-β1/Smad2/3 and Erk1/2 signaling	TGF-β1, Smad2/3	[132]
						cell (2, 5, 10 μmol/L)			
2					Silica-induced PF mice	Mice (3, 10 mg/kg)	Inhibition of ECM precipitate formation	GSH, HYP, MDA, IL-1β, IL-6, TNF-α, TGF-β1	[131]
3	Rhei radix et rhizoma	Polygonaceae	Purging and defecating, clearing heat and detoxification	Chrysophanol	BLM-induced PF mice	Mice (10 mg/kg)	Inhibits Wnt/β-catenin signaling	β-catenin, HYP, TNF-α, IL-1β, IL-6, IFN-γ	[226]
4				Rhapontin	BLM-induced PF rats and THP-1 cells	Rats (25, 50, 100 mg/kg)	Regulation of TGF-β/Smad signaling	TGF-β, Smad	[137]
						cell (50 μmol/L)			
5	Salviae miltiorrhizae radix et rhizoma	Lamiaceae	Promoting blood circulation and removing blood stasis, reducing swelling and relieving pain	Salvia miltiorrhiza	BLM-induced PF mice and NIH3T3 cells	Mice (21, 40, 80 mg/kg)	anti-oxidation	Nrf2, NOX4	[228]
						Cell (0.1, 1, 3 μmol/L)			
6				Tanshinone IIA	Silica-induced PF mice	Mice (25 mg/kg)	Inhibit TGF-β1/Smad signaling	TGF-β1, α-SMA, Smad2/3/4/7	[307]
7				Cryptotanshinone	BLM-induced PF rats and HLFs cells	Rats (7.5, 15, 30, 60 mg/kg)	Inhibition of TGF-β/Smad signaling	TGF-β1, HYP, TNF-α, IL-6, ColI, ColIII, α-SMA	[304]
						Cell (0.75–18 mg/mL)			
8	Astragalus membranaceus	Fabaceae	Replenishing qi and solidifying the surface, strengthening the upright and dispelling evil	Astragaloside IV	Silica-induced PF rats	Rats (20 mg/kg)	Inhibition of TGF-β/Smad3 signaling	TGF-β1, α-SMA, Collagen III, Smad2/3	[306]
						Cell (7, 20, 60 μg/mL)			
9					BLM-induced PF rats	Rats (10 mg/kg)	Inhibition of ECM precipitate formation	HMGB1, HYP, Col-III, LN, HA	[305]
10	Scutellaria baicalensis Georgi	Scutellaria	Clearing away heat and dampness, purging fire and detoxifying	Baicalein	MRC-5 cells	Cell (1, 10 μmol/L)	Downregulation of CTGF expression	CTGF, TGF-β1, Smad2/3	[308]
11	Curcumae longae rhizoma	Zingiberaceae	Promoting blood circulation and removing blood stasis, Regulating menstruation and relieving pain	Curcumin	BLM-induced PF rats	Rats (300 mg/kg)	Inhibit TGF-β1 signaling	TGF-β1, HYP, Collagen III	[309]
12					BLM-induced PF rats	Rats (300 mg/kg)	Inhibition of ECM precipitate formation	fibronectin, lung glycoproteins	[235]
13	Tripterygium wilfordii Hook. f	Ranunculaceae	Clearing heat and detoxification, promoting blood circulation and removing blood stasis	Triptolide	radiation-induced PF mice and NIH3T3 cells	Mice (0.25 mg/kg)	Inhibit NF-κB signaling	NF-κB, LOX, IκBα	[239]
						Cell (5 ng/mL)			
14				Isorhynchophylline	Silica-induced PF mice	Mice (20 mg/kg)	Anti-inflammatory	/	[240]
15	Rhodiolae crenulatae radix et rhizoma	Crassulaceae	Replenishing qi and activating blood circulation, dredging pulse and relieving asthma	Rutin	BLM-induced PF rats	Rats (50, 100 mg/kg)	Regulation of TGF-β1/α-SMA/Col I/III signaling	TGF-β1, α-SMA	[259]
16	Ginseng radix et rhizoma	Araliaceae	Replenish qi and nourish blood, invigorate body and quench thirst	Total ginsenoside	BLM-induced PF mice	Mice (40, 80, 160 mg/kg)	Regulation of TGF-β1/Smad signaling	TGF-β1, α-SMA, Smad2/3/7, MMP-2, MMP-9	[310]
17	Dioscorea polystachya Turczaninow	Dioscoreaceae	Tonifying spleen and lung, nourishing yin and moistening dryness	Dioscin	Silica-induced PF mice and AM MH-S cells	Mice (mg/kg)	Promotes autophagy in alveolar macrophages	LC3, p62, AKT, mTOR, BECN1	[311]
						Cell (200, 400, 800 nmol/L)			
18	Artemisia annua L	Asteraceae	Clear deficiency heat and remove bone steaming	Dihydromyricetin	BLM-induced PF mice and MLG cells	Mice (50, 100, 200 mg/kg)	Inhibit TGF-β1/Smad signaling	TGF-β1, α-SMA	[197]
19	Epimedii folium	Berberidaceae	Kidney deficiency and impotence, sore waist and knees	Icariin	BLM-induced PF rats	Rats (60 mg/kg)	Inhibit Hippo signaling	YAP, IL-1β, IL-6, TGF-β1, TNF-α	[295]
20	Chelidonii herba	Papaveraceae	Clearing heat and detoxification, reducing swelling and relieving pain	Chelerythrine	BLM-induced PF mice	Mice (0.375, 0.75 mg/kg)	activate Nrf2/ARE signal transduction	Nrf2, ARE	[244]
21	Psoraleae fructus	Fabaceae	Warm the kidney and consolidate the essence, strengthen the muscles and bones	Psoralen	BLM-induced PF mice and NIH3T3 cells	Mice (5 mg/kg)	Inhibition of ECM precipitate formation	TNF-α, IL‐1β	[167]
						Cell (5, 10, 20, 40 μmol/L)			
22	Citri reticulatae pericarpium	Rutaceae	Regulating qi and eliminating food, resolving phlegm and relieving cough	citrus alkaline extracts (CAEs)	BLM-induced PF mice	Mice (16, 32, 64 mg/kg)	Inhibit TGF-β1/Smad3 signaling	TGF-β1, LOX, HYP	[312]
23	Salviae miltiorrhizae radix et rhizoma/chuanxiong rhizoma	Lamiaceae/Apiaceae	Promoting blood circulation and removing blood stasis, Clearing heat and detoxification	Salvia miltiorrhiza and ligustrazine	BLM-induced PF rats	Rats (125 + 43.75, 250 + 87.5, 500 + 175 mg/kg)	Regulation of TGF-β/Smad signaling	TNF-α, TGF-β1	[313]
24	Rabdosiae rubescentis herba	Lamiaceae	Clearing heat and detoxification, reducing swelling and relieving pain	Oridonin	BLM-induced PF mice and MRC-5 cells	Mice (10, 20 mg/kg)	Regulation of TGF-β/Smad signaling	TGF-β, Smad	[314]
						Cell (5, 10 μmol/L)			
25	Curcumae longae rhizoma	Zingiberaceae	Promoting blood circulation and removing blood stasis, Regulating menstruation and relieving pain	curcumin/Curcumol	HFL cells	Cell (8, 16, 32 μg/mL)	Inhibition of ECM precipitate formation	Col-I, Col-III, TGF-β1, α-SMA	[315]
26	Citrus fruits	Rutaceae	Invigorate the spleen and replenish qi, moisturize the lungs and relieve cough	Hesperidin	BLM-induced PF rats	Rats (25, 50, 100 mg/kg)	Inhibits TGF-β1/Smad3/AMPK and I-κBα/NF-κB signaling	TGF-β1, NF-κB	[253]
27	Polygoni cuspidati rhizoma et radix	Polygonaceae	Clearing heat and detoxification, promoting blood circulation and removing blood stasis	Polydatin	BLM-induced PF rats and A549 cells	Rats (10, 40, 160 mg/kg)	Inhibit TGF-β1/Smad signaling	TGF-β1, Col-1, α-SMA, TNF-α, IL-6, IL-13	[156]
						Cell (0–120 μmol/L)			
28	Coptidis rhizoma	Ranunculaceae	Clearing heat and dryness, purging fire and detoxification	berberine	BLM-induced PF mice	Mice (50, 100, 200 mg/kg)	activate PPAR-γ	HGF, PPAR-γ	[260]
29	Astragali radix/ferulae resina	Fabaceae/Apiaceae	Replenishing qi and solidifying the surface, strengthening the upright and dispelling evil	astragaloside IV/ferulic acid	BLM-induced PF mice	Mice (24 + 40.8 mg/kg)	Inhibit TGF-β1/Smad3 signaling	TGF-β1, Nrf2	[261]
30	Centellae herba	Apiaceae	Clearing heat and detoxification, promoting diuresis and detumescence	Asiaticoside	BLM-induced PF mice	Mice (50 mg/kg)	Up-regulation of BMP7/Smad1/5 signaling	BMP7, Smad1/5	[263]
31	sophorae fla vescentis radix	Fabaceae	Clearing heat and detoxification, diuresis and purging gonorrhea	Matrine	MRC-5 cells	Cell (10 μmol/L)	Inhibit TGF-β/Smad2/3 signaling	TGF-β, Smad2/3	[182]
32	Blueberry	Ericaceae		Pterostilbene	A549 cells	Cell (0–100 μmol/L)	Inhibit TGF-β1 signaling	TGF-β1, Bcl-2, Bax, LC3, p62, p21	[183]
33	Siratia grosvenorii	Cucurbitaceae	Clear heat and moisturize the lungs and open pharynx	Mogrol	BLM-induced PF mice and NIH3T3 cells	Mice (1, 5, 10 mg/kg)	Regulation of TGF-β1 and AMPK signaling	TGF-β1, AMPK	[188]
						Cell (1, 5, 10 μmol/L)			
34	Rosmarinus officinalis	Lamiaceae		Rosmarinic Acid	radiation-induced lung damage rats	Rats (30, 60, 120 mg/kg)	Inhibits RhoA/Rock signaling	RhoA, Rock	[273]
35	Grape			Resveratrol	BLM-induced PF rats and MRC-5 cells	Rats (60 mg/kg)	Regulates MAPK/AP-1 signaling	MAPK/AP-1	[316]
						Cell (10 μmol/L)			
36	Kaempferiae rhizoma	Zingiberaceae	Promoting qi to relieve pain and regulating qi in a broad way	Alpha-Mangostin	BLM-induced PF mice	Mice (10 mg/kg)	Regulates AMPK signaling	AMPK, α-SMA, Smad2/3, Col I, MMP-9	[286]
37	Silybi fructus	Asteraceae	Clearing heat and detoxification, soothing the liver and promoting gallbladder	Silibinin	Silica-induced PF mice	Mice (100, 300 mg/kg)	Anti-inflammatory Inhibits EMT	MDA, GSH, HYP, IL-6, IL-1β, IL-17, TGF-β1	[196]
38	Pseudostellariae radix	Caryophyllaceae	Yiqi Jianpi, Shengjin fluid	Heterophyllin B	BLM-induced PF mice and MLE-12 cells	Mice (5, 20 mg/kg)	Activates AMPK, inhibits TGF-β1-Smad2/3 signaling, and downregulates STING	TGF-β1, CoL-1, α-SMA	[317]
						Cell (1–100 μmol/L)			
39	Arenaria kansuensis	Caryophyllaceae	Clearing heat and detoxification, diuresis and purging gonorrhea	A. kansuensis ethanol extract	paraquat-induced PF rats	Rats (170, 350, 700 mg/kg)	Inhibit NF-κB/TGF-β1/Smad2/3 signal transduction	NF-κB, TGF-β1, Smad2/3	[203]
40	Aurantii fructus immaturus	Rutaceae	Regulating qi stagnation, resolving phlegm and relieving cough	Neohesperidin	BLM-induced PF mice and NIH3T3, MLg, A549 cells	Mice (20 mg/kg)	Inhibition of TGF-β/Smad3 signaling	TGF-β1, Smad2/3, Erk, p-38, JNK	[208]
						Cell (0–200 μmol/L)			
41	Polyporus	Polyporaceae	Diuresis, detumescence and phlegm	Polyporus Polysaccharide	BLM-induced PF mice and HLF cells	Mice (50, 100 mg/kg)	Inhibit TGF-β1/Smad2/3 signaling	TGF-β1, Smad2/3	[210]
						Cell (1 mg/mL)			
42	Arnebiae radix	Boraginaceae	Clearing heat and detoxification, moistening dryness and invigorating muscle	Shikonin	MLFC cells	Cell (0.1, 0.3, 1, 3, 10 μmol/L)	Inhibit Akt signaling	MAPK, akt	[318]

### Mediate apoptosis and autophagy

Autophagy is the degradation of intracytoplasmic foreign bodies, damaged, and senescent cells by the cell's own structures via lysosomes, which helps maintain a homeostatic equilibrium between degradation and recirculation [319]. Cellular autophagy can promote PF by promoting fibroblast activation, myofibroblast differentiation, and ECM deposition, indicating that autophagy may be one of the key mechanisms in the pathogenesis of PF [320, 321]. The expression of cellular autophagy is typically deficient in all IPF lung tissues, which appears to be one of the risk factors for IPF, as suggested by the current study [322]. When autophagy is inhibited, lung fibroblast epithelial senescence and myofibroblast differentiation can be accelerated. Numerous studies have demonstrated that herbal medicine regulates cellular autophagy primarily via mTOR-dependent and -independent pathways. Apoptosis is a form of programmed cell death characterized primarily by cell shrinkage and the formation of apoptotic vesicles [323]. Excessive apoptosis of alveolar epithelial cells induces aberrant secretion of numerous cytokines, including TGF-β1, and accelerates the progression of PF [324]. Inadequate apoptosis of lung fibroblasts results in their massive transformation into myofibroblasts, which promotes ECM deposition and lung fibrosis [325]. There are multiple signaling pathways involved in the role of apoptosis in lung fibrosis. It has been hypothesized that the MAPK/NF-κB signaling pathway plays a crucial role in this.Autophagy and apoptosis are complex mechanisms that control cell growth and death under physiological and pathological conditions. It has been suggested that the two processes, reduced autophagic activity and apoptotic resistance, may be interrelated in IPF fibroblasts [326]. When mTOR activity is inhibited, autophagy increases, apoptosis increases, and resistance to apoptosis decreases. Enhanced autophagy is accompanied by increased apoptosis. Consequently, insufficient autophagy can result in insufficient apoptosis in fibroblastic cells and excessive apoptosis in alveolar epithelial cells, which, through a cascade of biological responses, leads to IPF.

Various components in the peel of citrus reticulata blanco have anti-inflammatory and antioxidant activities [327]. Wu’s experimental team extracted primary lung fibroblasts from normal mice and bleomycin induced PF mice for experiments. The results indicate that citrus alkaloid extract can effectively induce apoptosis in mouse lung fibroblasts, and its mechanism of action may be related to the p38/COX-2/Fas signaling pathway regulated by oxidative stress [328]. In addition, the experimental team explored the intervention effect of citrus alkaline extract on bleomycin induced PF in mice and its mechanism [329]. The experiment shows that citrine extract can effectively delay the degree of PF mice, and its mechanism may be through inhibiting NF-κB/p38-mediated signaling pathway inhibits cell apoptosis. In addition, studies have shown that citrus alkaloid extracts can activate COX-2 and β-Catenin/P53 pathway inhibits cellular senescence and reduces PF [330, 331]. To sum up, citrus extract can mediate cell apoptosis through multiple signaling pathways, but the specific pharmacodynamic substances in the extract that play a role in improving PF are still unclear, and further experimental research is needed.

Quercetin is a flavonol compound in TCM [332], which has anti-inflammatory, antioxidant, immunomodulatory and anti-tumor activities [333]. Literature studies have shown that quercetin alone cannot promote apoptosis, but quercetin can eliminate fibroblast resistance to apoptosis [334]. Further studies suggest that quercetin may enhance the susceptibility of aging fibroblasts to apoptosis by regulating the expression of caveolin-1 and Fas and activating AKT. In addition, Xiao found that quercetin not only promotes autophagy activity, but also improves fibrosis by inhibiting pro-fibrotic factors and AKT/mTOR signaling pathway [335]. These studies demonstrate quercetin's potential to combat PF by mediating apoptosis, adding to therapeutic strategies for IPF and other fibrotic diseases. However, these studies have only been conducted in vitro or in animals, and further clinical studies are still needed. In addition to the above-mentioned TCM that can improve PF, the detailed information of other TCM studies in the past five years that can improve PF through mediate apoptosis and autophagy were summarized and presented in Table 4.

**Table 4:** Details about some traditional Chinese medicine improving pulmonary fibrosis by mediating apoptosis and autophagy

No	Source	Genus information	Traditional efficacy	Active compound	Experiment model	Administration dose	Therapeutic target	cytokine	Refs.
1	Citri reticulatae pericarpium	Rutaceae	Regulating qi and eliminating food, resolving phlegm and relieving cough	citrus alkaline extracts (CAEs)	A549 cells	Cell (0.01 μmol/L)	Inhibit b-catenin/p53 signaling	PDGF, TNF-α, p21, p53, MMP-7, CTGF	[331]
2					BLM-induced PF mice and PMLF, MRC-5 cells	Mice (32, 64, 96 mg/kg)	prevent cellular senescence	COX-2, α-SMA, Fibronectin, p21, p16	[330]
						Cell (50, 100, 200 μmol/L)			
3					BLM-induced PF mice	Mice (16, 32, 64 mg/kg)	Inhibit p38/NF-κB signaling	p38, NF-κB	[329]
4					HPFS cells	Cell (50, 250, 500 μmol/L)	Induces apoptosis of lung fibroblasts	COX-2, Fas	[328]
5					BLM-induced PF mice and A549 cells	Mice (32, 64, 96 mg/kg)	Regulates PERK and ATF3/PINK1 signaling	PERK, ATF3, PINK1	[336]
						Cell (50, 100, 200 μg/mL)			
6	Curcumae longae rhizoma	Zingiberaceae	Promoting blood circulation and removing blood stasis, Regulating menstruation and relieving pain	Curcumin	LMSCs cells	Cell (2.5, 5, 10, 20 μmol/L)	Regulation of Akt/Nrf2/HO-1 signaling	Akt, Nrf2, HO-1	[234]
7					BLM-induced PF rats	Rats (300 mg/kg)	Inhibit TGF-β1 signaling	TGF-β1, HYP, Collagen III	[309]
8	Erigeron breviscapus	Asteraceae	Promoting blood circulation and removing blood stasis, clearing heat and detoxification	Scutellarein	BLM-induced PF mice	Mice (10 mg/kg)	Inhibits PI3K/Akt signaling	PI3K, Akt, Smad2/3, α-SMA	[172]
9	Amaryllidaceae	Amaryllidaceae	Dispel wind and detoxification, kill insects and stop itching	Lycorine	BLM-induced PF mice and BMDMs cells	Mice (5, 10, 20 mg/kg)	Inhibit the expression of NLRP3	NLRP3, MPO, IL-6, IL-1β, α-SMA	[288]
						Cell (0–40 mmol/L)			
10	Dioscorea polystachya Turczaninow	Dioscoreaceae	Tonifying spleen and lung, nourishing yin and moistening dryness	Dioscin	Silica-induced PF mice and AM MH-S cells	Mice (mg/kg)	Promotes autophagy in alveolar macrophages	MMP9,mtROS	[311]
						Cell (200, 400, 800 nmol/L)			
11	Blueberry	Ericaceae		Pterostilbene	LPS-induced PF mice	Mice (12.5, 25, 50 mg/kg)	Activation of Keap-1/Nrf2 inhibits A20/NF-κB and NLRP3 signaling	NF-κB, NLRP3	[268]
12	Various plant sources			Quercetin	Tracheal Trauma Rabbit and WI-38 cells	Cell (5–200 μmol/L)	Inhibits AKT/mTOR signaling	AKT, mTOR	[335]
13					BLM-induced PF mice	Mice (30 mg/kg)	Enhance fibroblast apoptosis	Fas, DR4/5, CAveolin-1, AKT	[334]
14				Sinapic acid	BLM-induced PF rats	Rats (10, 20 mg/kg)	Inhibit NF-κB/NRF2/HO-1 signaling	NF-κB, NRF2, HO-1	[250]
15	Bletillae rhizoma	Orchidaceae	Convergence to stop bleeding, reduce swelling and promote muscle growth	Bletilla striata	Silica-induced PF mice and A549 cells	Mice (20, 40 mg/kg)	Inhibit Bax/Bcl-2 signaling	HO-1, Nrf2, γGCSc, Bax, Bcl-2	[337]
						Cell (10, 25, 50 μmol/L)			
16	Atractylodis rhizoma	Asteraceae	Invigorating spleen and stomach, tonifying qi and blood	Atractylenolide III	BLM-induced PF rats	Rats (0.6, 1.2, 2.4 mg/kg)	activate Nrf2/NQO1/HO-1 signal transduction	Nrf2, NQO1, HO-1	[246]
17	Chuanxiong rhizoma	Apiaceae	Activating blood circulation and promoting qi, expelling wind and relieving pain	Ligustrazin	paraquat-induced PF mice and A549 cells	Mice (30 mg/kg)	Inhibit mTOR/Akt signaling	mTOR, Akt	[338]
18	Rhei radix et rhizoma	Polygonaceae	Purging and defecating, clearing heat and detoxification	Emodin	Silica-induced PF mice and A549 cells	Mice (25, 50, 100 mg/kg)	Regulation of NF-κB and TGF-β1/Smad3 signaling	NF-κB, TGF-β1	[136]
19	Salviae miltiorrhizae radix et rhizoma/puerariae lobatae radix	Lamiaceae/Fabaceae	Promoting blood circulation and removing blood stasis, reducing swelling and relieving pain	Tanshinone IIA/puerarin	BLM-induced PF mice and MRC-5 cells	Mice (5 + 14 mg/kg)	Inhibition of IL 6-JAK2-STAT3/STAT1 signaling	IL6, STAT1	[339]
20	Glycyrrhizae radix et rhizoma	Fabaceae	Nourishes qi and nourishes yin, clears away heat and detoxifies	Isoliquiritigenin	MRC-5 cells	Cell (0–40 mg/L)	Inhibit PI3K/AKT/mTOR signaling	PI3K, AKT, mTOR	[340]
21	Scutellariae radix	Scutellaria	Clearing away heat and dampness, purging fire and detoxifying	Baicalin	BLM-induced PF rats and RPF	Rats (50 mg/kg)	Regulation of CaMKII and PI3K/AKT signaling	PI3K, AKT	[141]
						Cell (20, 40, 60, 80 μg/mL)			
22	Rosmarinus officinalis	Lamiaceae/Fabaceae		Rosmarinic Acid + carnosic acid	BLM-induced PF rats and HLFs cells	Rats (5 + 3 mg/kg)	Induces apoptosis of lung fibroblasts	p21, AKT, p38	[341]
						Cell (50 + 10, 100 + 20 μmol/L)			
23	Cinnamomi cortex	Lauraceae	Warming the meridians and dispelling cold, dredging yang and reaching the camp	Trans-cinnamaldehyde	V79-4 cells	Cell (0–50 μmol/L)	Activates Nrf2/HO-1 signaling	Nrf2, HO-1	[284]
24	Rhizoma Kaempferiae	Zingiberaceae	Relieving pain by activating qi and dispelling cold in the middle of warming	Kaempferol	Silica-induced PF mice	Mice (150 mg/kg)	Anti-inflammatory	Beclin-1, p62, LC3, MMP-9, MMP-2	[342]
25	Schisandrae chinensis fructus	Magnoliaceae	Astringent and astringent, replenishing qi and invigorating fluid	Schisandra	BLM-induced PF rats	Rats (5 mg/kg)	Inhibit TGF-β1/Smad signaling	TGF-β1, Smad3/4/7	[343]
26	Leonuri herba	Lamiaceae	Promoting blood circulation and regulating menstruation, reducing swelling and relieving pain	Leonurine	BLM-induced PF mice	Mice (50, 100 mg/kg)	Up-regulation of AKT signaling	AKT, ECAD, TGF-β1, ACTA2, BAX	[294]
27	Ginkgo folium	Ginkgoaceae	Calming the liver and eyesight, moistening the lungs and relieving cough	Ginkgo biloba Extract EGb761	BLM-induced PF mice	Mice (25, 50, 100 mg/kg)	Regulation of NF-κB signaling	NF-κB, TNF-β, IL-1β, IL-6, α-SMA, TGF-β1	[344]

### Inhibition of endoplasmic reticulum stress

The endoplasmic reticulum (ER) is an organelle responsible for maintaining protein homeostasis in cells. The accumulation of misfolded proteins in the ER is referred to as ERS. Existing literature suggests that ERS is a key mechanism mediating PF in AEC [345, 346]. For example, ER stress promotes inflammation, induces EMT, and activates pro-apoptotic pathways, leading to the generation of PF [345]. In order to maintain homeostasis, cells rely on protective mechanisms to help them cope with ER stress, collectively known as the unfolded protein response (UPR). When the UPR mechanism fails or is over-activated, it can lead to cell apoptosis [347].

ERS can be triggered by various factors, including genetics, environmental exposure, viral infections, and cellular aging. Studies have demonstrated that ERS induced by genetic mutations, such as those found in surfactant protein C (SFTPC), plays a significant role in the development of PF [348, 349]. Additionally, environmental factors can also induce ERS, which is a critical factor in the pathogenesis of PF. For example, research has shown that environmental factors like silica and cigarette smoke can trigger ERS, and airborne particulate matter can activate signaling pathways like PERK and ATF, inducing ERS in cells [350]. Cigarette smoke can cause ERS in bronchial epithelial cells, leading to cell apoptosis in both in vitro and in vivo settings [351, 352]. Viral infections may contribute to fibrosis development by inducing ERS and activating UPR. Research has demonstrated that aging mice infected with herpes virus may develop PF through mechanisms related to ERS [353]. Additionally, aging can impair normal ER function in organisms [354, 355], which is in line with clinical observations indicating that PF is more prevalent in older individuals. In conclusion, enhancing protein processing and mitigating downstream effects of ERS may be an effective strategy to treat fibrosis resulting from ERS.

Citrus reticulata Blanco peel is used to treat lung diseases in Chinese Traditional medicine. Recently, Wang's team carried out a study on citrus extract to improve PF [336]. The results shown that citrus extract could reduce collagen deposition in PF mice and inhibit the increase of endoplasmic reticulum stress biomarkers. Further cell experiments shown that citrus extract regulated ERS through PERK and ATF3/PINK1 pathways. However, it is unclear whether citrus alkaline extract can improve PF by regulating ERS, which still needs further experiments. This study provides an important reference for the mechanism of citrus extract in improving PF.

Chlorogenic acid is the main active ingredient of many TCMs, which has antibacterial, antiviral, and free radical scavenging pharmacological effects. Wang's team evaluated the effect of chlorogenic acid on improving PF related markers through mice and cell models [356]. The results shown that chlorogenic acid can effectively regulate the expression of PF related pathological markers, and reduce the degree of PF by inhibiting the ERS pathway. At the same time, chlorogenic acid plays a certain role in regulating apoptosis involved in PF. Although this study did not deeply explore the signal pathway involved in chlorogenic acid inhibiting ERS, it provided a new experimental reference for chlorogenic acid to improve PF.

Isorhamnetin is a flavonoid active compound in hippophae fructus, which has pharmacological effects such as antioxidant, anti-inflammatory, and anti-tumor. Recent research literature suggests that isorhamnetin can effectively inhibit bleomycin induced collagen deposition and reduce type I collagen and α-SMA [194]. In addition, they further demonstrated that isorhamnetin can improve the degree of PF by inhibiting EMT and ERS. Although more studies are needed to clarify the mechanism of Isorhamnetin in improving PF, this study shown for the first time the potential important value of isorhamnetin in improving PF.

In recent years, the research of citrus alkaline extracts, isorhamnetin and chlorogenic acid to improve PF has made great progress. These studies have explored the activity and pharmacological mechanism of these compounds to improve PF in animal or cell experiments, making these compounds possible candidates for new drugs to treat PF. However, it should be pointed out that these studies have failed to delve into the association between ERS and inflammatory response, EMT, and cell apoptosis, and also lack in-depth research on signaling pathways and molecular mechanisms. Therefore, future research should pay more attention to exploring the molecular mechanism and signal pathway of TCM in improving PF, so as to better understand the pharmacology and toxicology of these candidate compounds. In addition to the above-mentioned TCM that can improve PF, the detailed information of other TCM studies in the past five years that can improve PF through inhibition of endoplasmic reticulum stress were summarized and presented in Table 5.

**Table 5:** Details about some traditional Chinese medicines improving pulmonary fibrosis by inhibiting endoplasmic reticulum stress

No	Source	Genus information	Traditional efficacy	Active compound	Experiment model	Administration dose	Therapeutic target	cytokine	Refs.
1	Citri reticulatae pericarpium	Rutaceae	Regulating qi and eliminating food, resolving phlegm and relieving cough	citrus alkaline extracts (CAEs)	BLM-induced PF mice and A549 cells	Mice (32, 64, 96 mg/kg)	Regulates PERK and ATF3/PINK1 signaling	PERK, ATF3, PINK1	[336]
						cell (50, 100, 200 μg/mL)			
2	Hippophae fructus	Elaeagnaceae	Invigorate the stomach and eliminate food, relieve cough and expectoration	Isorhamnetin	BLM-induced PF mice	Mice (10, 30 mg/kg)	Suppress EMT	PERK, α-SMA, Col III, CHOP, GRP78, TGF-β1	[194]
						Cell (25, 50, 100 μmol/L)			
3	Lonicerae japonicae flos	Caprifoliaceae	Clearing heat and detoxification, dispelling wind and detoxification	Chlorogenic acid	BLM-induced PF mice	Mice (15, 30, 60 mg/kg)	Inhibits endoplasmic reticulum stress	α-SMA, Col I, CHOP, PERK, IRE-1, GRP78	[356]

## Conclusion

PF is a prevalent lung disease, the prevalence and incidence of IPF increasing annually [6, 7]. If PF is not promptly and effectively treated, it will lead to a decline in lung function, which will have a negative impact on patients' quality of life and life expectancy [12]. In the last decade, glucocorticoids and immunosuppressants are commonly used in the treatment of PF, such as pirfenidone and nintedanib, but their efficacy is not ideal, they have many side effects, and they are expensive [14, 15]. Finding some reliable drugs from natural plants is becoming a research hotspot. TCM has been widely concerned because of its remarkable efficacy in treating or improving PF, mild side effects and low price. In the last five years, the scientific research on the improvement of PF by TCMs are increasing and has made great progress. Systematic and comprehensive summary of these research advances will help pharmaceutical researchers to understand the current research progress more quickly [16–18].

This article systematically reviews the progress in improving or reversing PF using traditional Chinese medicine in the last 5 years, and analyzes the major signaling pathways involved from a pharmacological perspective. Overall, the mechanism of improving PF mainly includes inhibiting EMT, anti-inflammatory and antioxidant, improving ECM deposition, mediating apoptosis and inhibiting ERS. The involved signaling pathways include TGF-β1/Smad, Nrf2/ARE, PI3K/AKT, NF-κB, etc. It is worth noting that the same Chinese medicine often involves multiple signaling pathways to improve pulmonary fibrosis, suggesting that these Chinese medicines have multi-target effects.

Although the experimental research of improving or treating PF with TCM has made some important progress, there are still some shortcomings. First, these experiments are mostly based on animal or cellular models and lack clinical trial validation. Secondly, most experiments often study only one or a few signaling pathways, lacking overall and comprehensive research. Third, because the pathogenesis of PF has not been fully elucidated, it also limits the depth of corresponding research in TCM, especially the toxicology research needs to be strengthened. What is most worth thinking about is how current pharmacological research can support the transformation of these TCM into new drugs.

On the basis of the existing known mechanisms of drug action, research methods based on artificial intelligence and big data computing are becoming the mainstream of drug development. The combination of computer aided drug design, drug molecular-target interaction, signaling pathway and pharmacological network, these new technologies will provide more than traditional research approaches to decrypt TCM treatment of PF, which will become a research hotspot in the near future.

## Data Availability

Not applicable.

## Abbreviations

PF: Pulmonary fibrosis
IPF: Idiopathic pulmonary fibrosis
ECM: Extracellular matrix
MAPK: Mitogen-activated protein kinase
IL: Interleukin
TNF-α: Tumor necrosis factor alpha
TGF-β: Transforming growth factor Beta
TIMPs: Tissue inhibitor of matrix metalloproteinases
ARE: Antioxidant response element
PI3K: Phosphoinositide 3-kinase
AKT: Protein kinase B
mTOR: Mammalian target of rapamycin
SASP: Senescence associated secretory phenotype
PDGF: Platelet-derived growth factor
TCM: Traditional Chinese medicine
EMT: Epithelial-mesenchymal transition
SAB: Salvianolic acid B
Tan-IIA: Tanshinone IIA
CPT: Cryptotanshinone
ROS: Reactive oxygen species
AG: Andrographolide
NOX: NADPH oxidase
TanIIA: Tanshinone IIA
ASV: Astragalus methyloside
Pts: Pterostilbene
ERS: Endoplasmic reticulum stress
AEC: Alveolar epithelial cells
ER: Endoplasmic reticulum
